# The horizontal gene transfer of *Agrobacterium* T-DNAs into the series *Batatas* (Genus *Ipomoea*) genome is not confined to hexaploid sweetpotato

**DOI:** 10.1038/s41598-019-48691-3

**Published:** 2019-08-29

**Authors:** Dora G. Quispe-Huamanquispe, Godelieve Gheysen, Jun Yang, Robert Jarret, Genoveva Rossel, Jan F. Kreuze

**Affiliations:** 1 Department of Biotechnology, Ghent University, 9000 Ghent, Belgium; 2 International Potato Center (CIP), Lima, Peru; 3 Shanghai Chenshan Plant Science Research Center, Chinese Academy of Sciences, Shanghai, China; 4 USDA-ARS/PGRU, Griffin, GA 30223 USA

**Keywords:** Speciation, Plant domestication

## Abstract

The discovery of the insertion of *Ib*T-DNA1 and *Ib*T-DNA2 into the cultivated (hexaploid) sweetpotato [*Ipomoea batatas* (L.) Lam.] genome constitutes a clear example of an ancient event of Horizontal Gene Transfer (HGT). However, it remains unknown whether the acquisition of both *Ib*T-DNAs by the cultivated sweetpotato occurred before or after its speciation. Therefore, this study aims to evaluate the presence of *Ib*T-DNAs in the genomes of sweetpotato’s wild relatives belonging to the taxonomic group series *Batatas*. Both *Ib*T-DNA1 and *Ib*T-DNA2 were found in tetraploid *I*. *batatas* (L.) Lam. and had highly similar sequences and at the same locus to those found in the cultivated sweetpotato. Moreover, *Ib*T-DNA1 was also found in *I*. *cordatotriloba* and *I*. *tenuissima* while *Ib*T-DNA2 was detected in *I*. *trifida*. This demonstrates that genome integrated *Ib*T-DNAs are not restricted to the cultivated sweetpotato but are also present in tetraploid *I*. *batatas* and other related species.

## Introduction

The sweetpotato [6X *Ipomoea batatas* (L.) Lam] is a member of the genus *Ipomoea*, the largest genus in the morning glory (Convolvulaceae) family. This family contains approximately 50 genera and more than 1,000 species. Over half of these species are concentrated in the Americas, where they are distributed as cultigens, medicinal plants and weeds^1^. Among the morning glories, *I*. *batatas* is the only species with an economic importance as a major food crop^2^, although *I*. *aquatica* is also cultivated and consumed as a leafy vegetable, mainly in South-East Asia. Series *Batatas* is a subdivision within the genus *Ipomoea*. This is a relatively young clade that diversified circa 12 million years ago^3^. This group includes the cultivated hexaploid sweetpotato [*I*. *batatas* (L.) Lam], the tetraploid (4x) sweetpotato *I*. *batatas* (L.) Lam^4^, and 13 other species considered to be the wild relatives of the cultivated sweetpotato. These wild relatives are *I*. *cordatotriloba*, *I*. *cynanchifolia*, *I*. *grandiflora*, *I*. *lacunosa*, *I*. *leucantha*, *I*. *littoralis*, *I*. *ramosissima*, *I*. *splendor sylvae* (previously named *umbraticola*), *I*. *tabascana*, *I*. *tenuissima*, *I*. *tiliacea*, *I*. *trifida* and *I*. *triloba*^5,6^. Members of the series *Batatas* are endemic to the Americas, except *I*. *littoralis* that is native to Madagascar, South and Southeast Asia, Australia, and the Pacific region^5^. The basic chromosome number of the series *Batatas* species is 2n = 2 ×  = 30. While most species are diploid (2x), several are tetraploid (4x) or hexaploid (6x)^7^. To avoid confusion, hereafter in the current text, the (6x) sweetpotato (*I*. *batatas*) will be referred to as *Ib*6x, the tetraploid form of *I*. *batatas* as *Ib*4x, and the combination of both as “the sweetpotato group”.

The sweetpotato is a crop native to the Americas and it was an important food crop for the Inca and Mayan cultures. Its origin and center(s) of genetic diversity have been proposed as somewhere between the Yucatan Peninsula of Mexico and the mouth of the Orinoco River in Venezuela^8,9^, Peru and Ecuador^9^. Papua New Guinea, Indonesia and the Philippines are suggested as secondary centers of diversity^10^. Today, sweetpotato is a major staple food in numerous tropical countries^11^. However, its botanical origin and details about its domestication remain under debate.

Several hypotheses have been put forward to explain the sweetpotato’s botanical origin. Nishiyama^12^ proposed, based on cytogenetical studies, that *Ib*6x could have originated from the diploid species *I*. *leucantha*, from which the tetraploid *I*. *littoralis* was derived through polyploidization. The hybridization between these two species could have produced *I*. *trifida*, which is suggested to have different ploidies. Further cross-pollinations between these wild species, followed by selection and domestication of interesting genotypes, could have produced the *Ib*6x. Based on morphological and cytogenetical data, two additional hypotheses were subsequently suggested. Shiotani^13^ suggested that *I*. *trifida* forms an autopolyploid complex, and that the cultivated *Ib*6x is derived from this group. Austin^8^ suggested that the cultivated sweetpotato was derived from a hybridization event between *I*. *trifida* and *I*. *triloba*. Other studies carried out using molecular markers (RFLP, RAPD and SSR)^14–16^, beta-amylase gene sequences^17^ and cytogenetic analysis^18^ supported a contribution of *I*. *trifida* to the cultivated sweetpotato genome.

Advances in DNA sequencing technologies have allowed the assembly of complex polyploid genomes, including that of the cultivated sweetpotato. Yang *et al*.^19^ identified six haplotypes based on the assembly of a monoploid genome (15 pseudo chromosomes). The phylogenetic analysis of these haplotypes permitted the authors to trace back the hexaploidization process of *Ib*6x giving rise to a new hypothesis on its origin. These authors^19^ suggested that the cultivated sweetpotato could have arisen from a cross between a tetraploid and a diploid progenitor. The most likely diploid progenitor is *I*. *trifida*, while the tetraploid progenitor is currently unknown. It is not unreasonable to suspect that *Ib*4x, described by Bohac *et al*.^20^; Jarret *et al*.^16^; Roullier *et al*.^21^, which are known to share haplotypes with *Ib*6x^22^, might be the tetraploid progenitor.

A more recent, but related, hypothesis about the origin of the cultivated sweetpotato has been proposed by Muñoz-Rodríguez *et al*.^23^. These authors, based on the phylogenetic analyses of nuclear and chloroplast DNA regions, have proposed that *Ib*6x has a monophyletic origin (by autopolyploidization) and suggested that *I*. *trifida* is its most probable progenitor. This hypothesis also indicated a second role for *I*. *trifida* in the origin of the sweetpotato. Once *Ib*6x arose from *I*. *trifida*, it expanded its distribution range further than *I*. *trifida*’s natural distribution. Over time, both species became reciprocally monophyletic and then hybridized, giving rise to two cultivated sweetpotato lineages.

These previous investigations suggest that a further study of *Ib*4x and their wild relatives in series *Batatas* is required since they are key in efforts to elucidate the botanical origin of the cultivated sweetpotato.

The discovery of *Agrobacterium Ib*T-DNA1 and *Ib*T-DNA2, inserted into the *Ib*6x genome constitutes a noteworthy example of an ancient HGT event in a domesticated crop^24^. *Ib*T-DNA1 contains genes for auxin biosynthesis (T_R_-T-DNA like), while *Ib*T-DNA2 contains *RolB/C* genes (T_L_-T-DNA like). The acquisition of these genes by the cultivated sweetpotato and other *Ipomoea* species opens the possibility that these sequences have played a role in the evolution of this crop and its related species^25^. However, whether the acquisition of one or both *Ib*T-DNAs by the *Ib*6x genome occurred before or after its speciation remains unknown. To address this issue, it is necessary to evaluate the presence/absence of *Ib*T-DNA1 and *Ib*T-DNA2 insertions in members of the sweetpotato group and/or other members of the series *Batatas*. The resulting knowledge might be expected to shed light on the botanical origin of the cultivated sweetpotato and also provide critical clues related to the time of the ancestral *Agrobacterium* infection(s). Hence, the current study proposes to evaluate (i) the presence of *Ib*T-DNA1 and *Ib*T-DNA2 in the sweetpotato group and other *Ipomoea* (series *Batatas*) species and (ii) the use of *Ib*T-DNA1 and *Ib*T-DNA2 genes as markers to reconstruct the evolutionary history of the sweetpotato.

## Results

### Distribution of *Ib*T-DNA1 and *Ib*T-DNA2 in *Ipomoea spp*. series Batatas

The presence of *Agrobacterium* T-DNAs (*Ib*T-DNA1 and *Ib*T-DNA2) in the genome of *Ib*6x was demonstrated by Kyndt *et al*.^24^. Likewise, a limited number of wild relatives, including *Ib*4x and member species of the series *Batatas*, were evaluated in that work. Nine *Ib*4x and four representatives of the species *I*. *triloba*, *I*. *tabascana* and *I*. *trifida* were tested for the presence of *Ib*T-DNA genes [*Acs*, *C-prot*, *iaaH*, *iaaM* and *ORF13* (Open Reading Frame 13)] by PCR, using sequence-specific primers. None of *Ib*T-DNA genes were detected in these samples except for the *ORF13* gene (on *Ib*T-DNA2) in *I*. *trifida*.

**Figure 1:**
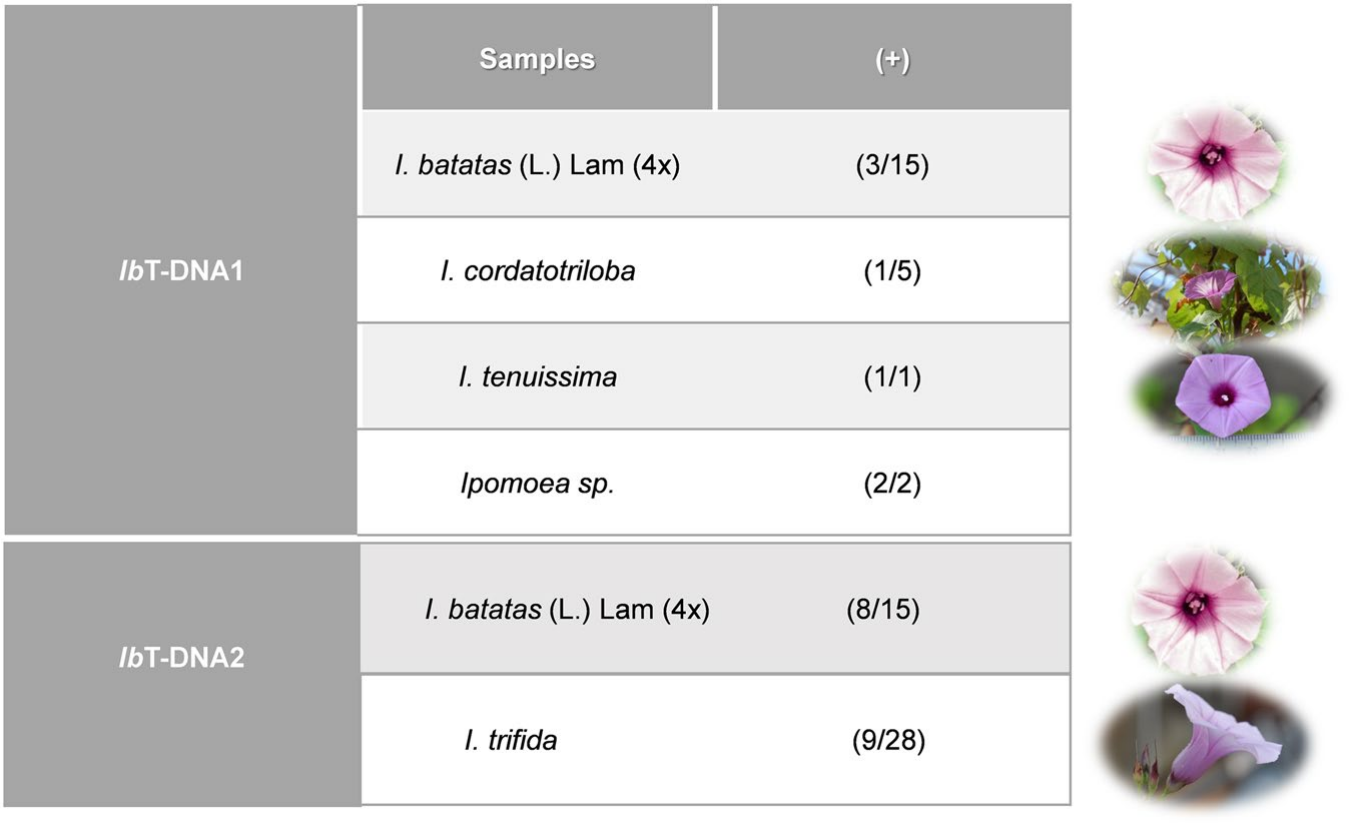
*Ib*T-DNA1 and *Ib*T-DNA2 detected in the wild relatives.

The current analysis was extended to include a total of 14 species representative of *Ipomoea* series *Batatas*, 2 species corresponding to other *Ipomoea* members (not in series *Batatas*) and 5 from related genera (Supplementary Data; Tables 1–4) using newly designed degenerate primers. *Ib*T-DNA1 genes were detected in *Ib*4x (3 out of 15) and 3 other species in the series *Batatas*, including; *I*. *cordatotriloba* (1 out of 5), *I*. *tenuissima* (1 out of 1) and one ambiguous *Ipomoea sp*. (2 out of 2). The *Ib*T-DNA2 gene was detected in 8 out of 15 *Ib*4x and 9 out of 28 *I*. *trifida* (Fig. 1). No other *Ipomoea* species outside of the series *Batatas* (0 out of 2) and no species from related genera (0 out of 5) examined in this study tested positive for the presence of *Ib*T-DNA genes by PCR using the degenerate primers.

**Figure 2:**
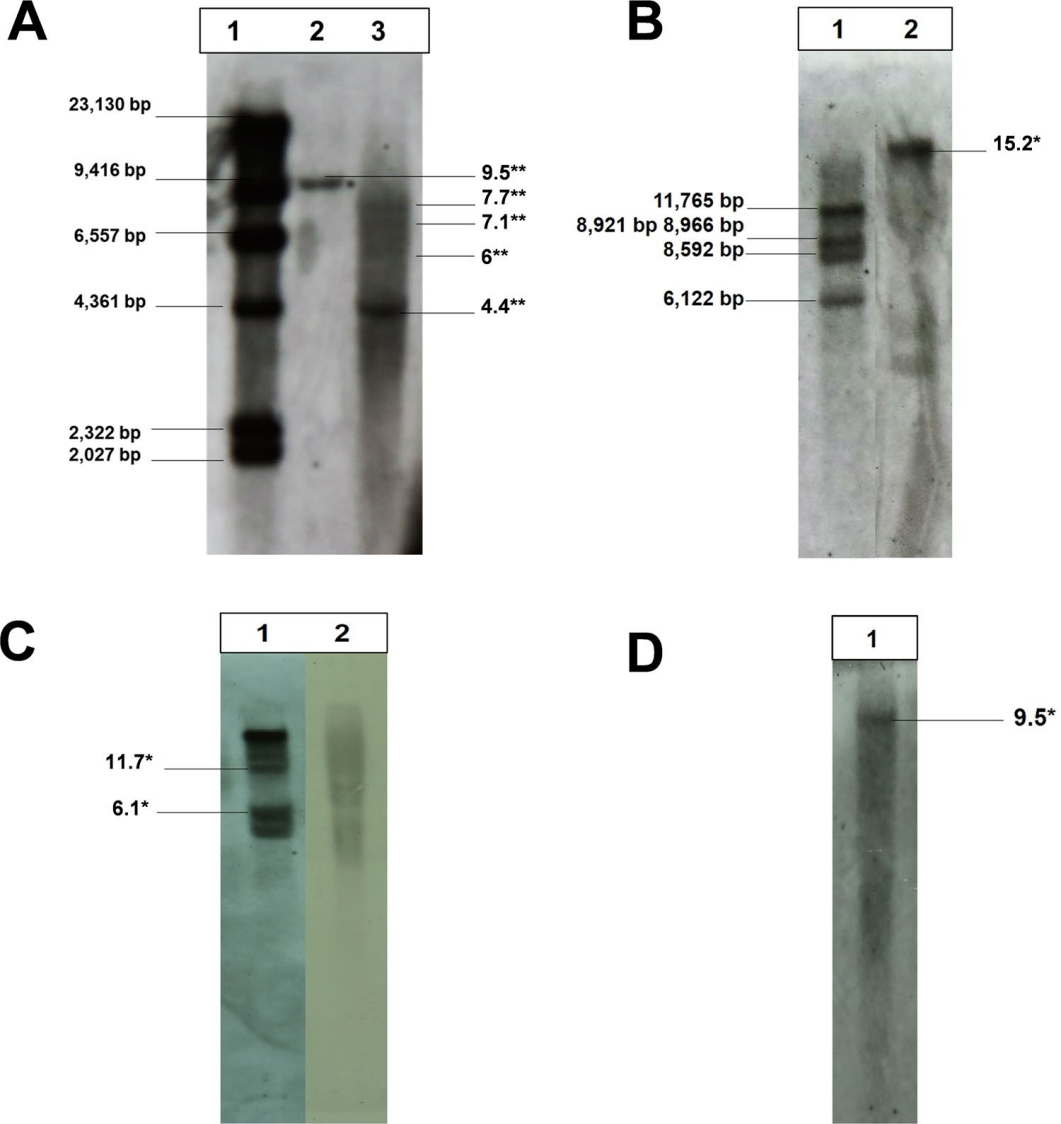
Southern blot with *Ib*T-DNA1 (*C-prot* probe, **A**–**C**) and *Ib*T-DNA2 (*ORF17n* probe, **D**) on *Spe* I digests of *Ipomoea spp*. series *Batatas*. (A1) DNA ladder; (A2) *I*. *batatas* (L.) Lam CIP 403270 (4x); (A3) *I*. *batatas* (L.) Lam *var*. *apiculata* PI 518474 (4x). (B1) *I*. *batatas* (L.) Lam cv. Huachano CIP 420065 (6x); (B2) *Ipomoea sp*. CIP 460250 (2x). (C1) *Ipomoea sp*. CIP 460814 (2x); (C2) *I*. *cordatotriloba* PI 518494 (2x). D1) *I*. *batatas* (L.) Lam PI 518474 (4x). In A3, sizes with ** were estimated from the DNA ladder. In B2, B3, C1, C2, D1, sizes with * were estimated from *I*. *batatas* (L.) Lam cv. Huachano CIP 420065.

The presence of *Ib*T-DNA1 was analyzed and confirmed by DNA blot analysis in two PCR positive *Ib*4x accessions (PI 518474 and CIP 403270) and the three PCR positive wild relatives (*Ipomoea sp*. and *I*. *cordatotriloba*). *Ipomoea batatas* (L.) Lam. var. *apiculata* (PI 518474) (Fig. 2A3) showed four bands - like *Ib*6x (Fig. 2B1); while CIP 403270 (*Ib*4x) showed only one (Fig. 2A2). *Ipomoea sp*. CIP 460250 (2x) displayed at least 1 band (Fig. 2B2), whereas *Ipomoea cordatotriloba* PI 518494 (2x) (Fig. 2C2) and *Ipomoea* sp. CIP 460814 (2x) (Fig. 2C1), appear to have at least four bands. The presence of *Ib*T-DNA2 was only tested and confirmed in *Ib*4x PI 518474 (1 band – Fig. 2D).

### Characterization of wild *Ipomoea* species

Phenotypic characterization (using ~30 descriptors, compiled based on Austin^26^ and Huamán^27^) confirmed the identity of accessions (Supplementary Data, Tables 2–4), with some exceptions. CIP 460250, which was collected as *I*. *trifida*, lacks the correct fruit and flower characteristics for the species. CIP 460397, collected as *I*. *tiliacea*, possesses flowers suggesting *I*. *trifida*. CIP 460786, collected as *I*. *grandifolia*, was morphologically similar to *I*. *cordatotriloba*. Conversely, CIP 460814 and CIP 460815 were collected as *I*. *cordatotriloba*, but had the characteristics of *I*. *grandifolia* (*I*. *grandifolia* and *I*. *cordatotriloba* are very similar, differing only in the size of the corolla, and some authors consider them varieties of the same species). CIP 460002 was collected as *I*. *leucantha*, which is a hybrid species between *I*. *trichocarpa* and *I*. *lacunosa* and which has highly variable characteristics. CIP 460811 was collected as *I*. *cordatotriloba*, however its flower color is white rather than violet as is typical for *I*. *cordatotriloba*.

### Phylogeny of *Ib*T-DNA1 and *Ib*T-DNA2 genes among *Ipomoea* species

**Figure 3:**
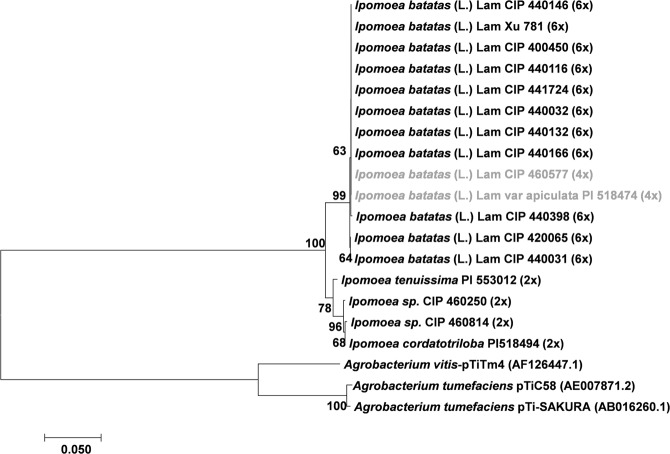
Phylogenetic tree generated by Neighbor-Joining of *C-prot* (827 nt) alignment. Values at the nodes show percentage of bootstrap support (of 1,000 bootstrap replicates) and they are indicated if greater than 50. Accession numbers (CIP/PI) and ploidy level are indicated for *Ipomoea* spp. whereas plasmid names are indicated for *Agrobacterium spp*. GenBank accession numbers are provided between brackets when available. Tetraploids (4x) *I*. *batatas* (L.) Lam are highlighted in grey.

**Figure 4:**
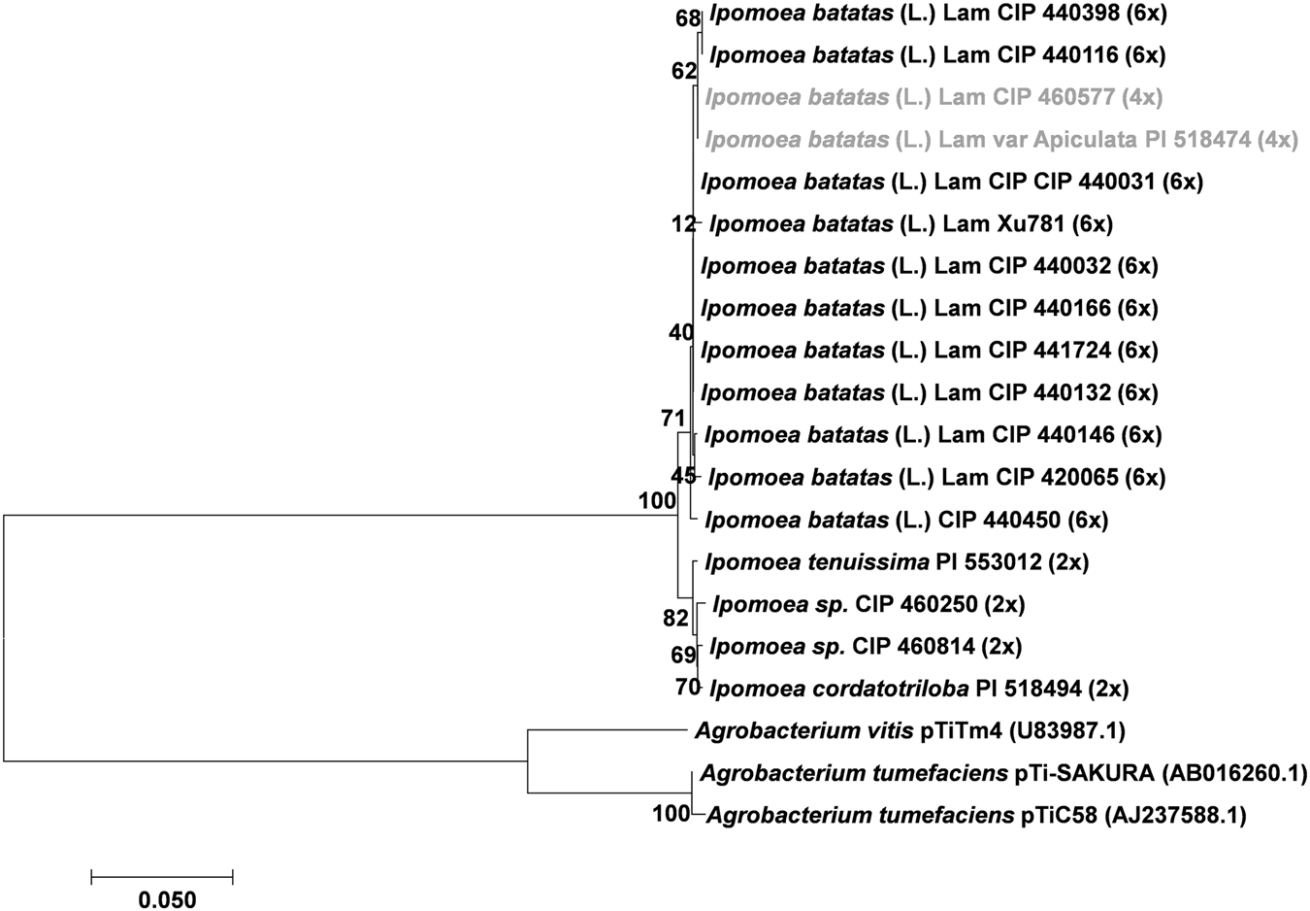
Phylogenetic tree generated by Neighbor-Joining of Acs (792 nt) alignment. Values at the nodes show percentage of bootstrap support (of 1,000 bootstrap replicates) and they are indicated if greater than 50. Accession numbers (CIP/PI) and ploidy level are indicated for *Ipomoea spp*. whereas plasmid names are indicated for *Agrobacterium spp*. GenBank accession numbers are provided between brackets when available. Tetraploids (4x) *I*. *batatas* (L.) Lam are highlighted in grey.

**Figure 5:**
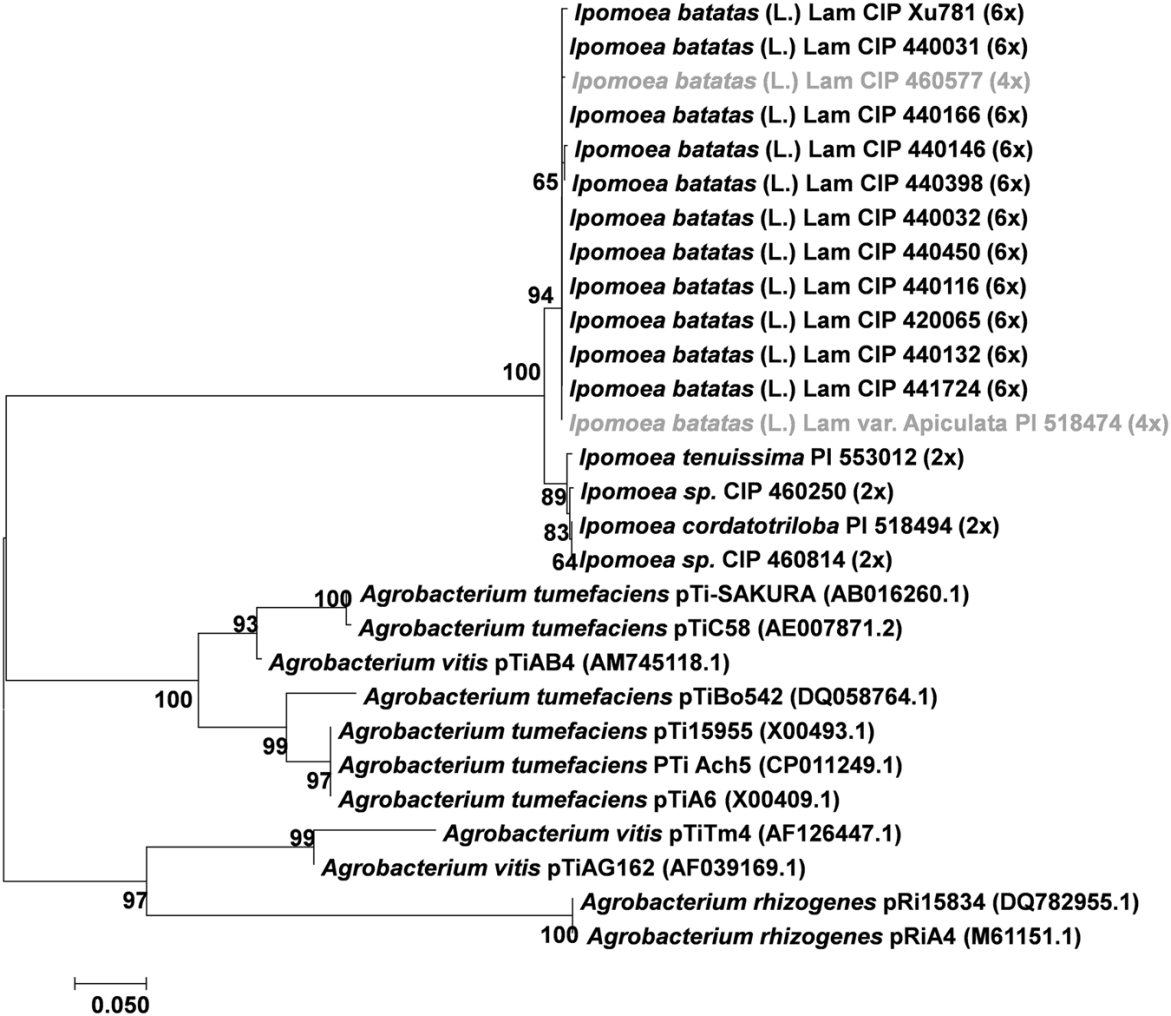
Phylogenetic tree generated by Neighbor-Joining of *iaaH* (641 nt) alignment. Values at the nodes show percentage of bootstrap support (of 1,000 bootstrap replicates) and they are indicated if greater than 50. Accession numbers (CIP/PI) and ploidy level are indicated for *Ipomoea* spp. whereas plasmid names are indicated for *Agrobacterium spp*. GenBank accession numbers are provided between brackets when available. Tetraploids (4x) *I*. *batatas* (L.) Lam are highlighted in grey. *Ipomoea sp*. CIP 430434 was previously labeled as tetraploid (4x) *I*. *batatas* (L.) Lam.

**Figure 6:**
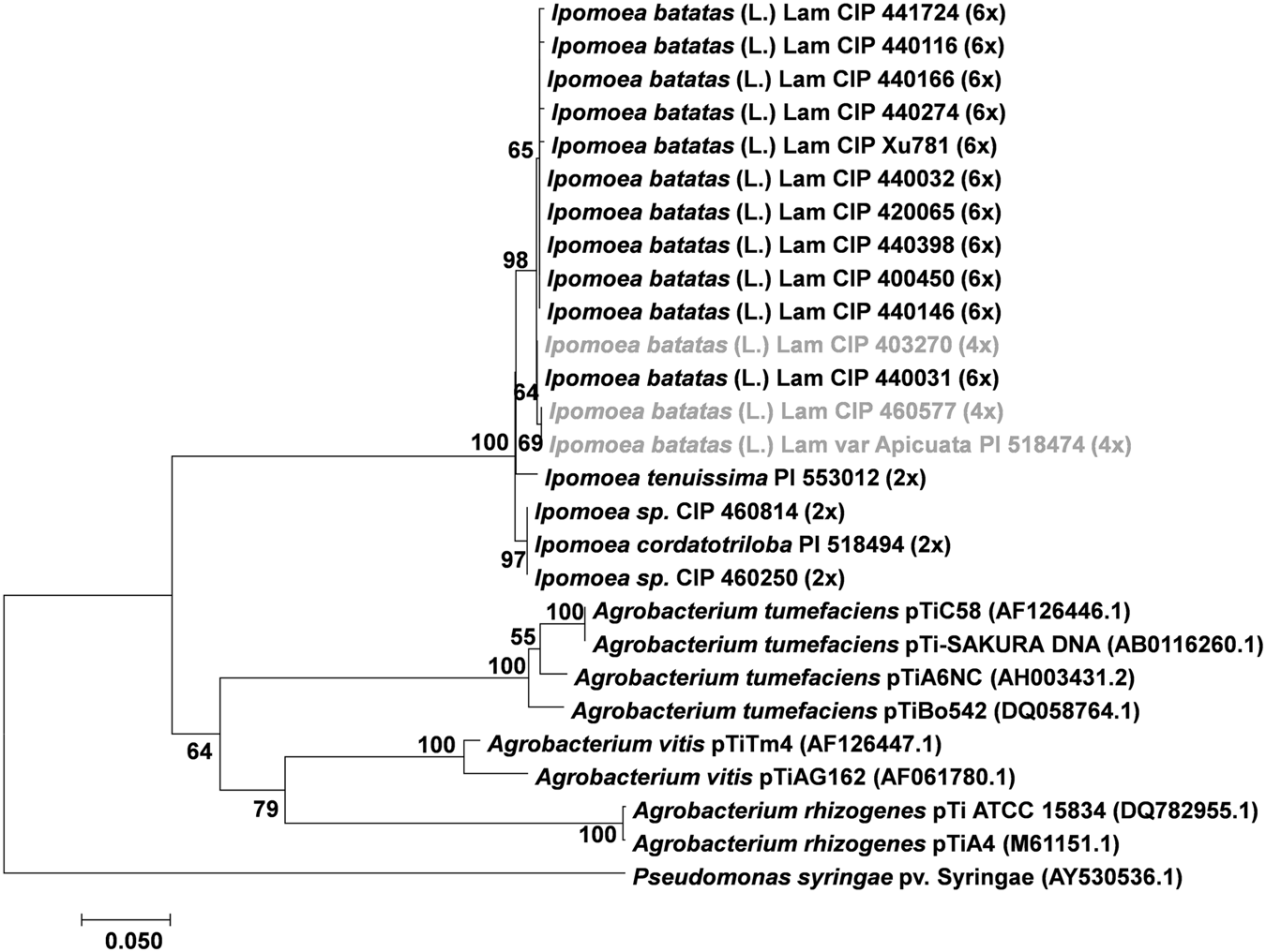
Phylogenetic tree generated by Neighbor-Joining of *iaaM* (485 nt) alignment. Values at the nodes show percentage of bootstrap support (of 1,000 bootstrap replicates) and they are indicated if greater than 50. Accession numbers (CIP/PI) and ploidy levels are indicated for *Ipomoea spp*. whereas plasmid names are indicated for *Agrobacterium spp*. GenBank accession numbers are provided between brackets when available. Tetraploids (4x) *I*. *batatas* (L.) Lam are highlighted in grey.

**Figure 7:**
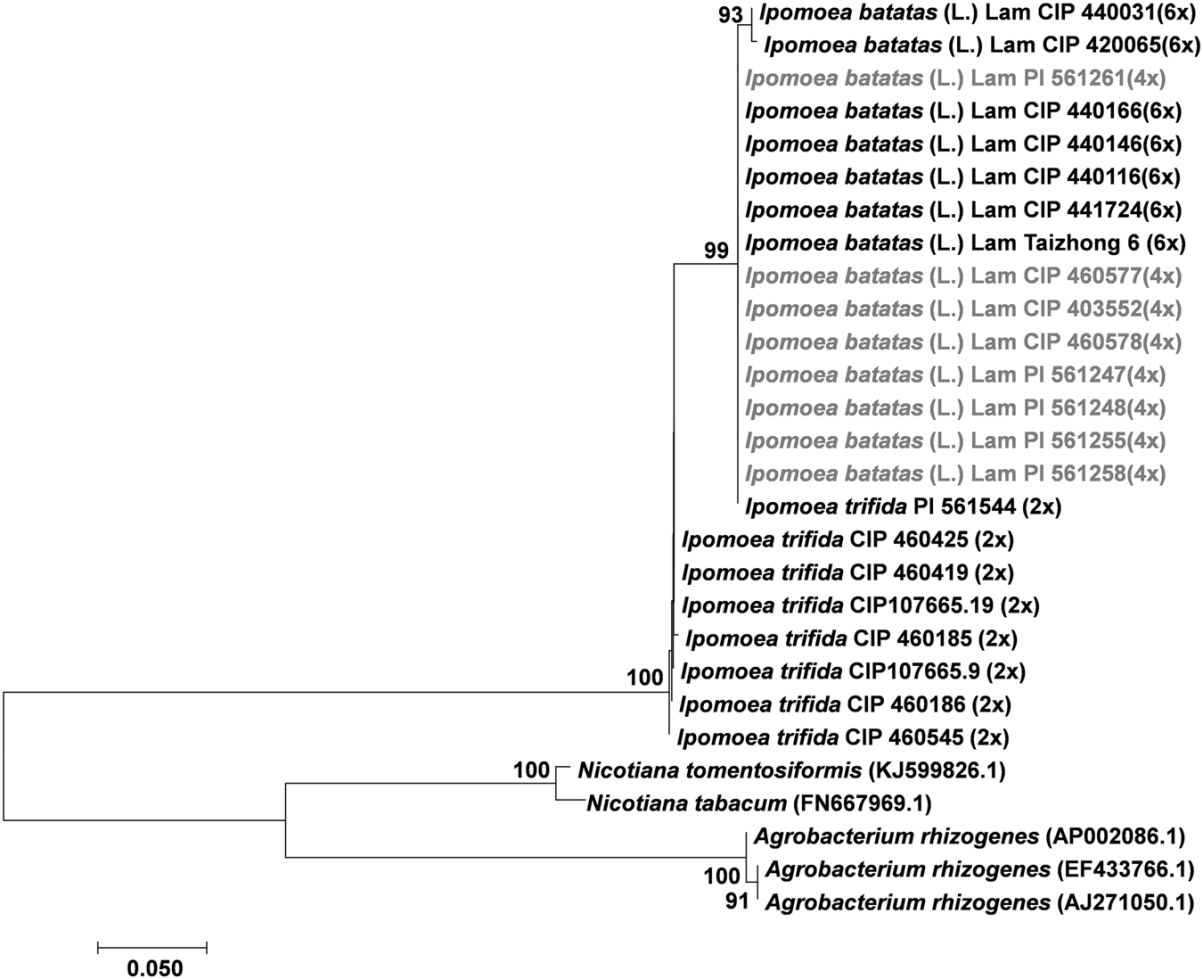
Phylogenetic tree generated by Neighbor-Joining of *ORF13* (492 nt) alignment from *Ib*T-DNA2. Values at the nodes show percentage of bootstrap support (of 1,000 bootstrap replicates) and they are indicated if greater than 50. Accession numbers (CIP/PI) and ploidy level are indicated for *Ipomoea* spp. GenBank accession numbers are provided between brackets for *Agrobacterium spp*. *and Nicotiana spp*. Tetraploids (4x) *I*. *batatas* (L.) Lam are highlighted in grey.

Phylogenetic analyses were performed to determine how *Ib*T-DNA sequences are related in the genus *Ipomoea* (Figs 3–7). Four phylogenetic trees were inferred using the *Ib*T-DNA1 genes *C-prot* (827 nt; Fig. 3), *Acs* (792 nt; Fig. 4), *iaaH* (641 nt; Fig. 5), and *iaaM* (485 nt; Fig. 6). The results obtained consistently showed that the *Ib*6x and *Ib*4x accessions group together (bootstrap value 71–99%), with the wild relatives as a sister clade [*Ipomoea sp*. (2 out of 2), *I*. *cordatotriloba* (1 out of 5) and *I*. *tenuissima* (1 out of 1)]. Both groups, *Ib*6x and *Ib*4x and their wild relatives, form a monophyletic group as compared to homologous genes from other sequenced T-DNAs; suggesting that they belong to the same lineage with a common origin.

In the case of the *Ib*T-DNA2 *ORF13* gene (492 nt; Fig. 7), the analysis indicates that *Ib*6x and *Ib*4x accessions grouped together in a well-supported clade (bootstrap value 99%) that includes one *I*. *trifida* accession PI 561544. The rest of the *I*. *trifida* samples formed a basal group and together with the sweetpotato group, they form a well-supported lineage (bootstrap value = 100). Nucleotide sequences from two species of the genus *Nicotiana* were included in the analysis of *Ib*T-DNA2. The results show that those are phylogenetically closer to *A*. *rhizogenes* strains pRi2659 (AJ271050.1), K599 (EF433766.1) and MAFF03-01724 (AP002086.1) in comparison with the *Ipomoea* sequences.

### *Ib*T-DNA1 and *Ib*T-DNA2 gene similarities among *Ib*6x and its wild relatives

**Figure 8:**
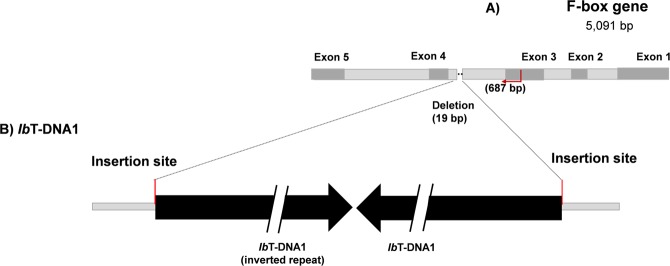
*Ib*T-DNA1 insertion in *F-box* gene. (**A**) A schematic representation of F-box gene (Taizhong 6) showing their 5 exons; a deletion (19 bp) in the target site is represented as dot lines among exon 3 and 4. (**B**) *Ib*T-DNA1 (Xu 781); *Ib*T-DNA1 and its inverted repeat are presented as interrupted black arrows. The region flanking *Ib*T-DNA1, to be analyzed in the next section (Fig. 7), is indicated as red arrows and its size (687 bp) is placed between brackets.

Pairwise comparisons of identities of partial nucleotide sequences of *Ib*T-DNA1 genes (*C-prot*, *Acs*, *iaaH*, *iaaM*) and *Ib*T-DNA2 gene (*ORF13*) were estimated. Nucleotide sequence identity values are above 99% for all genes analyzed within the sweetpotato group; which includes both *Ib*6x and *Ib*4x. Of note is that *ORF13* from *Ipomoea trifida* PI 561544 shows higher identity values (~99.9%) with the sweetpotato group than the rest of the *Ipomoea trifida* accessions (Supplementary Data, Tables 6 and 7). Among the sweetpotato group and its wild relatives, the identity values of all genes analyzed ranged from 96–98.8%. Previously, *Ib*T-DNA1 was found to be inserted in two copies, in the form of a partial inverted repeat, in the genome of the *Ib*6x cv. Xu781^24^. In the present study, the nucleotide sequence identity between the two copies of *Ib*T-DNA1 (Fig. 8) was calculated in Xu781, which corresponded to 98.8% (divergency 1.2%).

### *Ib*6x and *Ib*4x share the same insertion site of *Ib*T-DNA1

**Figure 9:**
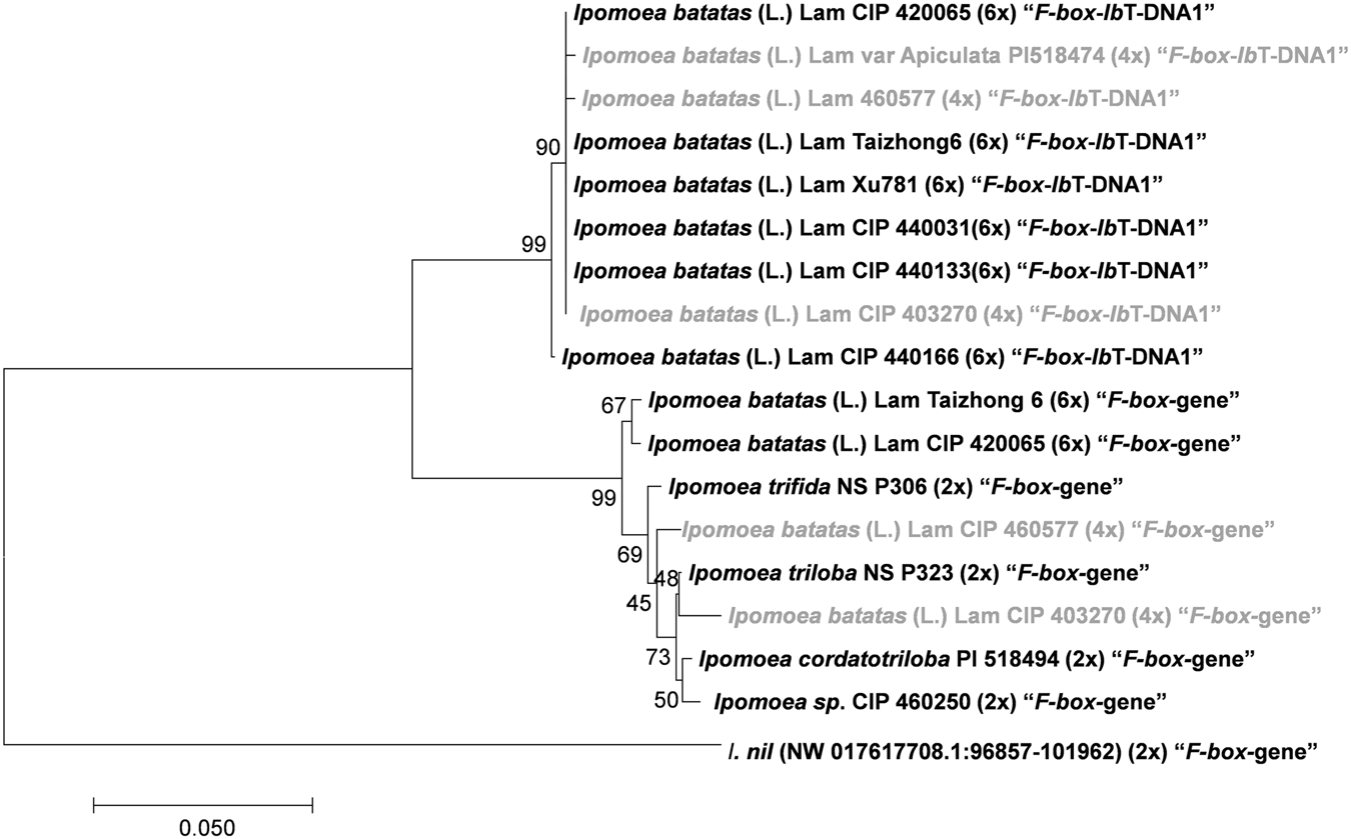
Phylogenetic tree generated by Neighbor-Joining of the *F-box* gene intact and containing *Ib*T-DNA1 (687nt, *F-box* third intron) Values at the nodes show percentage of bootstrap support (of 1,000 bootstrap replicates) they and are indicated if greater than 50. Accession numbers (CIP/PI) and ploidy level are indicated for *Ipomoea spp*. *F-box* gene interrupted by *Ib*T-DNA1 is indicated as “*F-box*-*Ib*T-DNA1”, whereas *F-box* gene without *Ib*T-DNA1 is labeled as “F-box gene”. Tetraploids (4x) *I*. *batatas* (L.) Lam are highlighted in grey. The GenBank accession number is provided between brackets for *I*. *nil*.

A phylogenetic analysis of the region flanking *Ib*T-DNA1 (687 nt; *F-box* third intron) was performed in order to elucidate the evolutionary relationship among all accessions in the sweetpotato group carrying *Ib*T-DNA1 (Fig. 9). The alignment included: *F-box*-*Ib*T-DNA1 sequences of six *Ib*6x accessions and three *Ib*4x accessions; *F-box* gene (without *Ib*T-DNA1) of two *Ib*6x and three *Ib*4x; and *F-box* gene of the wild relatives *I*. *trifida*, *I*. *triloba*, *I*. *cordatotriloba* and *Ipomoea sp*. CIP 460250. An *F-box* gene sequence from *I*. *nil*, cv. Tokyo-kokei, were included as an outgroup. The resulting tree shows that the *Ib*6x and *Ib*4x *F-box* genes carrying *Ib*T-DNA1 group together in a well-supported clade (bootstrap value = 99%). Likewise, sequences corresponding to the *F-box* gene uninterrupted by *Ib*T-DNA1 appear in a sister clade. This suggests that the *F-box* gene carrying *Ib*T-DNA1 might have diverged from the original *F-box* gene (either before or after the T-DNA insertion or both) and that the *Ib*6x and the *Ib*4x belong to the same lineage with a common origin. The nucleotide sequence identity calculated between *F-box* intact and *F-box*-*Ib*T-DNA1 was 96.9% (3.1% divergence). The regions flanking *Ib*T-DNA1 from *I*. *tenuissima*, *I*. *cordatotriloba* and *Ipomoea sp*. could not be included in the analysis since we were unable to amplify them with the primers designed.

### Analysis of *Ib*T-DNA2 in cultivated sweet potato Taizhong 6

**Figure 10:**
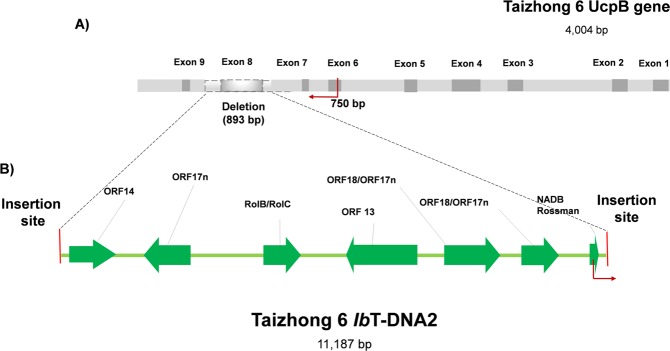
*Ib*T-DNA2 insertion sites in *UcpB* gene. (**A**) A deletion of 893 bp is indicated as a grey box with dot lines between 7^th^ and 8^th^ introns. (**B**) *Ib*T-DNA2 of cv. Taizhong 6, including ORFs with significant homology to *ORF18/ORF17n, ORF13, RolB/RolC* family, *ORF17n, ORF14* and a hypothetical protein with a “NADB Rossman” domain. Insertion sites are indicated as red lines; DNA filler as dark blue boxes at both ends. The region flanking *Ib*T-DNA2, to be analyzed in the next section (Fig. 10), is indicated as red arrows and its size (750 bp) is shown.

The region flanking *Ib*T-DNA2 in the *Ib*6x genome has not been described previously. It was predicted based on whole-genome sequencing data from cv. Taizhong 6. This analysis indicated that *Ib*T-DNA2 (cv. Taizhong 6) is inserted in chromosome 7 and has an estimated size of 11,187 bp (Fig. 10). It comprises seven open reading frames (ORFs) homologous to *ORF18/ORF17n*, *ORF13*, *RolB/RolC* family, *ORF17n*, *ORF14* and a hypothetical protein with a “NADB Rossman” domain of *Agrobacterium rhizogenes*. Compared to *Ib*T-DNA2 in cv. Huachano (KM052617), there is an insertion of 369 bp within *ORF13* cv. Taizhong 6. The region flanking *Ib*T-DNA2 was confirmed using PCR, and on the basis of significant homology (via tblastx) it was identified as the mitochondrial substrate carrier family protein *UcpB* - the highest score associated with *Ipomoea nil* (e-value = 6e-108; score = 1494). There is also an uninterrupted copy of the *UcpB* gene (without *Ib*T-DNA2) on chromosome 7 of cv. Taizhong 6, that is 4,004 bp in size with nine exons. The insertion site of *Ib*T-DNA2 was determined by comparing *UcpB* and *UcpB*-*Ib*T-DNA2. On one side, the T-DNA is flanked by an intronic region with high A/T-content after exon 7 while the other side is located in an intronic region 24 bp upstream from exon 9. Linked to the T-DNA insertion, there is a deletion of 893 bp in the *UcpB* gene that includes exon 8 (Fig. 10).

### *Ipomoea trifida*, *Ib*6x and *Ib*4x share the same *Ib*T-DNA2 insertion site

**Figure 11:**
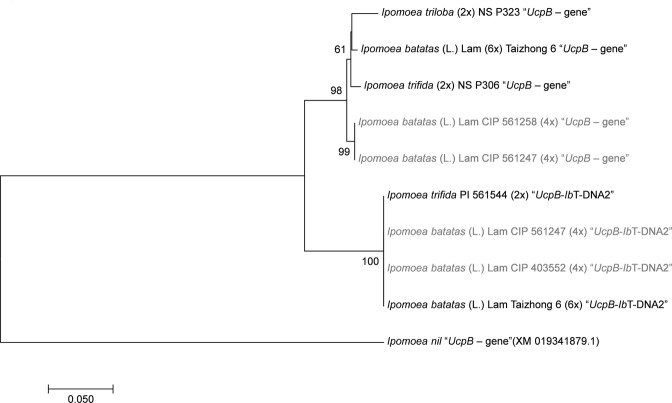
Phylogenetic tree generated by Neighbor-Joining of the *UcpB* gene intact and containing *Ib*T-DNA2 (750 nt, six intron– seven exon) Values at the nodes show percentage of bootstrap support (of 1,000 bootstrap replicates) and they are indicated if greater than 50. Accession numbers (CIP/PI) and ploidy level are indicated for *Ipomoea spp*. *UcpB* gene interrupted by *Ib*T-DNA2 is indicated as “*UcpB*-*Ib*T-DNA1”, whereas *UcpB* gene without *Ib*T-DNA2 is labeled as “*UcpB* gene”. Tetraploids (4x) *I*. *batatas* (L.) Lam are highlighted in grey. The GenBank accession number is provided between brackets for *I*. *nil*.

A phylogenetic analysis of the region flanking *Ib*T-DNA2 (750 nt, sixth intron – seventh exon) was performed in order to elucidate the evolutionary relationship between *UcpB* genes, with and without *Ib*T-DNA2 (Fig. 11). The alignment included: *UcpB*-*Ib*T-DNA2 sequences from one *Ib*6x, two *Ib*4x, one *I*. *trifida*, and *UcpB* gene sequences (without *Ib*T-DNA2) from one *Ib*6x, two *Ib*4x, one *I*. *trifida* and one *I*. *triloba*. A *UcpB* sequence from *I*. *nil*, cv. Tokyo-kokei, was included as an outgroup. The resulting tree shows that *Ib*6x, *Ib*4x and *I*. *trifida UcpB* sequences carrying *Ib*T-DNA2, group together in a well-supported clade (bootstrap value = 100%). Likewise, sequences containing only the *UcpB* gene (without *Ib*T-DNA2) appear in a sister clade. In addition, the nucleotide sequence identity between *ucpB* and *UcpB*-*Ib*T-DNA2 was estimated 95.7% (divergency 4.3%).

## Discussion

Our data demonstrate that the HGT event of *Agrobacterium* into series *Batatas* taxa is not confined to the hexaploid sweetpotato. It is present also in its wild relatives, which includes its tetraploid form, as well as other members of the series *Batatas*. We report here the detection of sequences homologous to *Ib*T-DNA1 and *Ib*T-DNA2 genes in at least ten accessions corresponding to *Ib*4x and fourteen accessions belonging to *I*. *trifida*, *I*. *cordatotriloba*, *I*. *tenuissima*, and a currently unidentified *Ipomoea sp*. from the series *Batatas*. Accessions belonging to the genus *Ipomoea*, but not members of series *Batatas*, and other related genera, were also analyzed. These included members of the *Quamoclit* group and species from the genera *Calystegia*, *Xenostegia*, *Operculina* and *Merremia*. The presence of *Ib*T-DNA1 and *Ib*T-DNA2 could not be confirmed in any of these samples. However, it should be noted we cannot exclude the possibility of false negatives in our analyses, and our findings likely represent an underestimation of the HGT events across the target species. This is because despite using degenerate primers and Southern blots, only regions corresponding to a few genes were tested, and remnants of (re-arranged) T-DNAs may exist that do not contain these complete regions. Also, we generally only tested one or two seedlings from each wild *Ipomoea* sp. accession (which are maintained as seeds) and if the accession was segregating for T-DNAs their presence could have been missed by chance.

The tetraploid form of *I*. *batatas* has been poorly characterized and its taxonomic status remains unclear. This taxon, collected from Ecuador, Colombia, Guatemala and Mexico, has been a subject of interest for over 50 years. The fact that these samples form thickened “pencil-shaped” storage roots has been considered as evidence that the tetraploids are primitive sweetpotatoes^28^. Some accessions were initially tentatively identified as *I*. *trifida* but later they were classified as wild *I*. *batatas*^20^. Subsequently, it was observed that the tetraploid form shared haplotypes (based on chloroplast and nuclear DNA markers) with the cultivated hexaploid^21^. These findings reinforced the hypothesis proposed by several authors, who suggested that tetraploid *I*. *batatas* are the closest wild relative of the cultivated sweetpotato^21,29^.

In the current study, nucleotide sequence analyses (pairwise comparisons) of *Ib*T-DNA1 and *Ib*T-DNA2 genes reveal high identity values (above 99%) among accessions from the sweetpotato group (*Ib*6x and *Ib*4x). These results were supported by the phylogenetic analyses of the regions flanking *Ib*T-DNA1 and *Ib*T-DNA2, which showed that *Ib*6x and *Ib*4x share the same insertion site (Figs 9 and 11). These findings reinforce previous taxonomic and molecular studies^20,21^ and suggest that *I*. *batatas* includes both hexaploid and tetraploid forms. However, there is also a possibility that the tetraploid form represents an interspecific hybrid between *I*. *batatas* and a close wild relative (*I*. *trifida*). We suggest the use of *Ib*T-DNA1 and *Ib*T-DNA2 genes as markers to further elucidate the origin of the sweetpotato in a manner similar to the use of *Agrobacterium* T-DNAs to reconstruct the evolution of *Nicotiana* and *Linaria*^30^.

The series *Batatas* contains the sweetpotato group and 13 other species considered to be its closest wild relatives^5,6^. Within this group, the species *I*. *trifida* has been identified as a potential wild ancestor in several studies based on morphological data, molecular markers and cytogenetic analyses^14–18^. Recently, two studies have reopened the debate about the role of *I*. *trifida* in the origin of the sweetpotato. Yang *et al*.^19^ analyzed a complete 6x *I*. *batatas* genome and proposed that the crop species could have resulted from a cross between a tetraploid and a diploid (most likely *I*. *trifida*) progenitor. Such a hybridization would have resulted in triploid progeny that, subsequently undergoing genome duplication, would result in 6x forms. In contrast, Muñoz- Rodriguez *et al*.^23^, based on genomic analyses of whole chloroplast and single-copy nuclear DNA regions, proposed that *I*. *trifida* played a dual role in the origin of the cultivated sweetpotato. Firstly, to form the first *I*. *batatas* lineage, as its most likely progenitor by autopolyploidization and, secondly, as the species that this autopolyploid (6x) later hybridized with to produce another independent sweetpotato lineage. Most recently, Wu *et al*.^31^ found through sequence comparison of the genome of hexaploid *I*. *batatas* with the genomes of *I*. *trifida* and *I*. *triloba*, that approximately one third of the hexaploid *I*. *batatas* genome shows higher similarity to *I*. *triloba* than to *I*. *trifida*. In relation to the data in the present study, the detection of *Ib*T-DNA2 (*ORF13* gene) only in the *I*. *trifida* accessions (9 out of 28) examined, and not in the other series *Batatas* species examined, provides additional evidence supporting the close relationship of this species (*I*. *trifida*) with the hexaploid and tetraploid forms of *I*. *batatas*. Furthermore, the phylogenetic analysis of *Ib*T-DNA2 and its flanking region indicated that *I*. *batatas* (6x and 4x) and *I*. *trifida* originated from a common ancestor.

Similar to the cT-DNAs in *Nicotiana* species^30^, it is possible that *Ib*T-DNA2 was acquired initially by *I*. *trifida* (or a common ancestor of *I*. *trifida* and *I*. *batatas*) and later transmitted across speciation events to the sweetpotato. This hypothesis is reinforced by the fact that *I*. *trifida*, together with *Ib*6x and *Ib*4x, share the same insertion site of *Ib*T-DNA2. An alternative explanation for the presence of *Ib*-TDNA2 in the sweetpotato involves its transfer by interspecific hybridization that is known to occur between *I*. *batatas* and *I*. *trifida*^21^. *Ipomoea trifida* accessions carrying the *ORF13* gene do not form a monophyletic group as PI 561544 appears in the clade of the sweetpotato group. This accession was collected in Venezuela and could represent the closest sweetpotato wild relative, in addition to the tetraploid form of *I*. *batatas*.

Species from the series *Batatas* other than *I*. *trifida* have also been proposed as potential contributors to the origin of the sweetpotato, albeit these hypotheses are less generally accepted within the community. Jarret *et al*.^16^ considered *I*. *tabascana* (4x), *I*. *trifida* and K233 (4x, suggested to be a hybrid between *I*. *batatas* and *I*. *trifida*) to be the closest relatives of the cultivated sweetpotato based on RFLPs, among the taxa examined (which did not include *Ib*4x). Recently, Eserman^32^ concluded, based on hybridization analysis, that *Ib*6x could have hybrid ancestry, with parentage from *I*. *ramosissima* and either *I*. *triloba* or *I*. *cordatotriloba*. The present study indicates the presence of *Ib*T-DNA1 genes in accessions belonging to the species *I*. *cordatotriloba*, *I*. *tenuissima* and two as yet unclassified *Ipomoea accessions* (CIP 460250 and CIP 460814). Our phylogenetic trees of *Ib*T-DNA1 genes indicate that the sweetpotato group, *I*. *cordatotriloba*, *I*. *tenuissima* and *Ipomoea* sp. form a strongly supported (~99% bootstrap) monophyletic clade as compared to their homologues in *Agrobacterium spp*., suggesting a common ancestry. The identity of the two *Ipomoea sp*. accessions containing *Ib*T-DNA1 has not been elucidated. These accessions were initially classified as *I*. *trifida* (CIP 460250) and *I*. *cordatotriloba* (CIP460814). However, upon morphological re-evaluation, it became clear that they were not consistent with the recorded classification. The latter shows phenotypic characteristics consistent with *I*. *grandifolia*, whereas the formers’ characteristics are not consistent with any of the established species. This was also confirmed by molecular markers, which showed CIP 460250 formed a sister clade compared to other *Ipomoea* series batatas^33^. It is not clear to what extent, if any, mis-identification of plant materials may have clouded efforts to resolve relationships within this group of taxa.

The presence of *Ib*T-DNA1 in *Ib*4x, *I*. *cordatotriloba*, and other *Ipomoea spp*. from the series *Batatas* was confirmed by southern blot analyses. Tetraploid *I*. *batatas* (CIP403270 and PI 518474) and wild relatives (*Ipomoea sp*. and *I*. *cordatotriloba*) show dissimilar banding patterns when compared to *Ib*6x. Additionally, the identity values of *Ib*T-DNA1 genes, among the sweetpotato group members and the wild relatives, range between 96–98.8% which is lower than within the sweetpotato group (above 99%). Thus, if the T-DNAs found in the series *Batatas* spp. represent a single ancestral event, it indicates that *Ib*T-DNA1 sequences have evolved and diverged since their acquisition by the sweetpotato’s ancestors. Recently, *Ipomoea* evolutionary trees have been calibrated, with an estimated mutation rate of 0.7% base pairs per million years^19^. The divergency between the repeats of *Ib*T-DNA1 is 1.2%, which leads to an estimated age of *Ib*T-DNA1 of 1.7 million years. Muñoz-Rodríguez *et al*.^23^ pointed out that the clade including the sweetpotato and *I*. *trifida* diverged from its sister clade at least 1.5 million years ago. Considering that *Ib*T-DNA1 is estimated to be older than the clade containing the sweetpotato and its potential ancestor (*I*. *trifida*); it is possible that *Ib*T-DNA1 might have been acquired early in the evolution of these species. Consequently, *Ib*T-DNA1 was fixed in the course of the evolution of the sweetpotato; while in other wild relatives it became less common, and in *I*. *trifida* this region could have been lost completely. The fact that *I*. *trifida* samples analyzed in this study do not contain *Ib*T-DNA1, supports this possible course of events.

Based on the current data, at least two hypotheses arise to explain the combined origin of *Ib*T-DNA1 and *Ib*T-DNA2 in the hexaploid *I*. *batatas*. Hypothesis I suggests that the HGT from *A*. *rhizogenes* (or an ancestral related species) may have occurred in a single event, transferring both *Ib*T-DNAs into a common ancestor of the species *I*. *trifida*, *I*. *tenuissima*, *I*. *cordatotriloba* and *I*. *triloba*. Subsequently, both regions were passed (independently or in combination) to *I*. *trifida*, *I*. *tenuissima*, *I*. *cordatotriloba* and *I*. *triloba* (or primitive forms). Later, one of these potential progenitors passed *Ib*T-DNAs to the tetraploid *I*. *batatas* (L.) Lam by speciation, which later became *I*. *batatas* (L.) Lam (6x). Hypothesis II proposes that the HGT from *Agrobacterium spp*. into the cultivated sweetpotato’s ancestor might have occurred via two or more independent events. It is possible that at least two species independently acquired *Ib*T-DNA1 and/or *Ib*T-DNA2 and then two of them combined in the common ancestor of *I*. *batatas* (L.) Lam (4x) and (6x). This hypothesis could explain the fact that the flanking region of *Ib*T-TDNA1 in *I*. *tenuissima* and *I*. *cordatotriloba* could not be amplified, despite using various sets of primers. Future efforts to determine the flanking sequences in these accessions should be able to confirm or discard this hypothesis. Nevertheless, based on our current data, because HGT events that enter the host germline are relatively rare in nature, and because of the clear correspondence between the phylogeny of the T-DNA genes and the species taxonomy, hypothesis I seems the most likely.

## Material and Methods

### Plant materials

In total, 114 plant samples were included in the present study. Detailed information on the accessions is included in Supplementary data (Tables 1–4). The materials included 11 accessions of hexaploid *Ipomoea batatas*, 15 accessions belonging to tetraploid *Ipomoea batatas* (4x), 82 accessions encompassing 13 species of the series *Batatas*, 2 accessions from other *Ipomoea* sp. (not series *Batatas*) and 5 accessions corresponding to related genera. The series *Batatas* species were distributed (numbers within parenthesis are the number of accessions sampled, within species) as: *Ipomoea trifida* (28), *I*. *triloba* (14), *I*. *cordatotriloba* (5), *I*. *grandifloria* (5), *I*. *tiliacea* (8), *I*. *ramosissima* (7), *I*. *leucantha* (5), *I*. *tabascana* (1), *I*. *tenuissima* (1), *I*. *littoralis* (1) and *I*. *splendor-sylvae* (2), *I*. *lacunosa* (1), *I*. *cynanchifolia* (1), unverified *Ipomoea sp*. (2). The other *Ipomoea* spp. examined (not series Batatas) are *I*. *heredifolia* (1) and *I*. *quamoclit* (1). *Ipomoea-*related species (other genera) included *Merremia quinquefolia* (1), *Merremia dissecta* (1), *Calystegia longipipes* (1), *Xenostegia tridentata* (1) and *Operculina aequisepala* (1). The plant materials were provided by the germplasm collection of the International Potato Center (CIP, Lima, Peru) and The National Genetic Resources Program (NGRP, USDA, USA).

For taxonomic verification, tetraploid *I*. *batatas* accessions from CIPs Genebank (3 siblings per accession) were germinated in a petri dish and then transferred to planting trays (Jiffy 7) for 15 days after which they were transferred into screenhouses for characterization using 30–60 descriptors (Rossel *et al*., unpublished). To determine the ploidy levels, samples from young leaves were analyzed in an Accuri C6 flow cytometer (BD Biosciences) with propidium iodide and data were analyzed with BD Accuri C6 Software. This was supplemented by chromosome counting in squashed root-tips stained with aceto-orcein as required.

### DNA sequences from other sources

Published DNA sequences from five *Ipomoea spp*. were added to our nucleotide alignment and analyses; including those derived from the genome browsers of cv. Taizhong 6 (http://ipomoea-genome.org/), *I*. *trifida* NSP306 and *I*. *triloba* NSP323 (http://sweetpotato.plantbiology.msu.edu/). The last two of these do not contain *Ib*T-DNA genes. Genebank and BAC library (KM113766) nucleotide sequences (KM113766), belonging to cv. Xu 781 and *I*. *nil* (XM 019334701.1 and XM 019341879.1), were also aligned and analyzed. *I*. *nil* does not contain *Ib*T-DNA genes and was used as an outgroup.

### DNA extraction

Laboratory procedures detailed below were performed essentially as previously described^24^. DNA extraction from fresh leaf tissues of 115 samples was performed using the CTAB method^34^. DNA quantity and quality were measured using a Nanodrop spectrophotometer (Thermo Fisher Scientific Inc., Waltham, MA, USA) and agarose gel electrophoresis, respectively.

### Screening for *Ib*T-DNAs in *Ipomoea spp*

Detection of *Ib*T-DNA1 and *Ib*T-DNA2 genes in *Ipomoea* samples was carried out by PCR using primers listed in Supplementary data (Table 5). The degenerate primers were designed for each gene (*Acs*, *C-prot*, *iaaH*, *iaaM* and *ORF13*) manually by examining multiple alignments of the target sequences from *Agrobacterium spp*. and 6x *I*. *batatas* (Supplementary Data Table 5.2). Part of the *Ipomoea*-specific malate dehydrogenase gene (MDH) was amplified from each DNA sample as a positive PCR control. The detection of the chromosome regions flanking *Ib*T-DNA1 and *Ib*T-DNA2 were carried out in *Ipomoea spp*. containing these regions by PCR. Likewise, uninterrupted *F-box* and *ucpB* genes were amplified by PCR. The PCR specific primers are listed in Supplementary data (Supplementary Data Table 5.3). PCR reactions were accomplished in 25-µl volumes containing 1x PCR buffer (Invitrogen, Carlsbad, CA, USA), 0.4 mM each of dGTP, dATP, dTTP, and dCTP; 0.3 µM of forward and reverse primer; 1 Unit of Taq DNA polymerase (Invitrogen); and 100 ng of genomic DNA. The PCR conditions were 94 °C for 5 min, followed by 35 cycles of 95 °C for 30 s, 50°–60 °C for 30 s, and 72 °C for 2 min, and then a final extension at 72 °C for 10 min. PCR products were separated on 1% agarose gels for visual detection of DNA.

### Sequencing and sequence analyses

PCR products were recovered using the Wizard SV gel extraction kit (Promega) according to the manufacturer’s recommendation. The eluted DNA was ligated into plasmid vector pCR 2.1 (Invitrogen), according to the manufacturer’s instructions, and cloned in *Escherichia coli* strain DH5α. PCR products were sequenced by LGC genomics, using the Sanger method and then assembled using the software Seqman II (DNAstar, Inc. Madison, WI, USA). Sequence alignments, phylogenetic analyses and pairwise comparison were performed using the software MEGA 5^35^.

### *Ib*T-DNA2 annotation

The flanking region of *Ib*T-DNA2 in the *Ib*6x genome was predicted based on whole-genome sequencing data from cv. Taizhong 6. *Ib*T-DNA2 in Taizhong 6 was annotated based on the top hits when performing blastn searches in the genome browser http://ipomoea-genome.org/.

### Southern blot hybridization

Southern blot analyses were performed to confirm previous PCR data on selected *Ipomoea* samples. Two probes complementary to the ORF coding for *C-protein* and *ORF17n* were utilized for these assays.

A total of 30 µg of genomic DNA was digested with *Spe I*, separated on a 0.8% agarose gel under 25 eV for 18 h, and transferred to a Hybond-N+ nylon membrane (Amersham Pharmacia Biotech, Piscataway, NJ, USA) with transfer buffer (20x SSC).

Primers used to amplify the DNA probes *C-prot* and *ORF17n* are listed in Supplementary Data (Table 5.4). Probe labeling was performed using the PCR DIG Probe Synthesis Kit (Roche, West Sussex, UK). Pre-hybridization and hybridization steps were carried out using the buffer DIG Easy Hyb (Roche), according to the manufacturer’s instructions. Following hybridization, membranes were washed twice (5 min) at low stringency (2x SSC, 0.1% SDS) at room temperature and two additional times (15 min) at high stringency (0.1x SSC, 0.1% SDS) at 65 °C. The images were captured by chemiluminescence on photosensitive film (Fujifilm Life Science).

## Supplementary information

Supplementary tables

## Data Availability

All data generated or analysed during this study are included in this published article (and its Supplementary Information Files). Sequence data have been deposited in GenBank database under accession number provided in the Supplementary Materials Table.
