# Supplementary material for: The horizontal gene transfer of Agrobacterium T-DNAs into the series Batatas (Genus Ipomoea) genome is not confined to hexaploid sweetpotato
Source: Sci Rep. 2019 Aug 29;9:12584. doi: 10.1038/s41598-019-48691-3 (PMC6715720; doi:10.1038/s41598-019-48691-3)
Supplement: Supplementary file 1 — Supplementary tables [file 41598_2019_48691_MOESM1_ESM.docx]

**Supplementary information for: The horizontal gene transfer event of *Agrobacterium* T-DNAs into the series *Batatas* (Genus *Ipomoea*) is not confined to hexaploid sweetpotato**

Dora G. Quispe-Huamanquispe, Godelieve Gheysen, Jun Yang, Robert Jarret, Genoveva Rossel and Jan F. Kreuze.

Contains supplementary Tables 1-8

**Table N°1 List of cultivated sweet potatoes (6x) that were sequenced to do the phylogenetic analyses of *Ib*T-DNA1 and *Ib*T-DNA2 genes**

Presence or absence was determined using the PCR reaction with degenerate primers (Table N° 6.1 and 6.2) for four ORFs on *Ib*T-DNA1 and one (*ORF13*) on *Ib*T-DNA2. ND indicates the test was not performed on the corresponding accessions.

| **Accession number** | **Accession name** | **Country of origin** | **Genus** | **Species** | **Ploidy**  **level** | **PCR MDH (Positive control)** | **PCR**  ***C-prot*** | **PCR *Acs*** | **PCR *IaaM*** | **PCR *IaaH*** | **PCR *ORF13*** |
| --- | --- | --- | --- | --- | --- | --- | --- | --- | --- | --- | --- |
| CIP 420065 | Huachano | Peru | *Ipomoea* | *batatas* | 6X | + | + | + | + | + | + |
| CIP 440132 | Beauregard | USA | *Ipomoea* | *batatas* | 6X | + | + | + | + | + | - |
| CIP 440166 | Tanzania | Uganda | *Ipomoea* | *batatas* | 6X | + | + | + | + | + | + |
| CIP 440031 | Jewel | USA | *Ipomoea* | *batatas* | 6X | + | + | + | + | + | + |
| CIP 440450 | Bogotana | Colombia | *Ipomoea* | *batatas* | 6X | + | + | + | + | + | - |
| CIP 440146 | CMR 1592 | Cameroon | *Ipomoea* | *batatas* | 6X | + | + | + | + | + | + |
| CIP 440116 | Gokoku-imo | Japan | *Ipomoea* | *batatas* | 6X | + | + | + | + | + | + |
| CIP 441724 | Cuitzeo | Mexico | *Ipomoea* | *batatas* | 6X | + | + | + | + | + | + |
| CIP 440398 | 500 (PI 308201) | New Zealand | *Ipomoea* | *batatas* | 6X | + | + | + | + | + | - |
| CIP 440032 | IITA-TIB 10 | Nigeria | *Ipomoea* | *batatas* | 6X | + | + | + | + | + | ND |
| Xu 781 |  | China | *Ipomoea* | *batatas* | 6X | ND | + | + | + | + | ND |
| Taizhong 6 |  | China | *Ipomoea* | *batatas* | 6X | ND | ND | ND | ND | ND | + |

**Table N°2 List of wild sweet potatoes (4x) that were tested for the presence of IbT-DNA1 and IbT-DNA2 genes using degenerate primers**

Presence or absence was determined using the PCR reaction with degenerate primers (Table N° 6.1 and 6.2) for four ORFs on *Ib*T-DNA1 and one (*ORF13*) on *Ib*T-DNA2. ND indicates the test was not performed on the corresponding accessions. Positive samples were sequenced.

| **Accession number** | **Collecting number** | **Country of origin** | **Genus** | **Species** | **Ploidy**  **Level** | **PCR MDH**  **(Positive control)** | **PCR Dg**  ***C-prot*** | **PCR Dg *Acs*** | **PCR Dg *IaaH*** | **PCR Dg *IaaM*** | **PCR Dg *ORF13*** |
| --- | --- | --- | --- | --- | --- | --- | --- | --- | --- | --- | --- |
| CIP 403248 | Col 1818 | Colombia | *Ipomoea* | *batatas* | 4X | + | - | - | - | - | - |
| CIP 403261 | Col 1843 | Colombia | *Ipomoea* | *batatas* | 4X | + | - | - | - | - | - |
| CIP 403270 | Col 1874 | Colombia | *Ipomoea* | *batatas* | 4X | + | - | - | - | + | - |
| CIP 403552 | Ecu 5283 | Ecuador | *Ipomoea* | *batatas* | 4X | + | - | - | - | - | + |
| CIP 403969 | Mex 2949 | Mexico | *Ipomoea* | *batatas* | 4X | + | - | - | - | - | - |
| CIP 460576 | UME 31 | Ecuador | *Ipomoea* | *batatas* | 4X | + | - | - | - | - | - |
| CIP 460577 | UME 32 | Ecuador | *Ipomoea* | *batatas* | 4X | + | + | + | + | + | + |
| CIP 460578 | UME 33 | Ecuador | *Ipomoea* | *batatas* | 4X | + | - | - | - | - | + |
| PI 561255 | Ecu 5296 | Ecuador | *Ipomoea* | *batatas* | 4X | + | - | - | - | - | + |
| PI 561258 |  | Ecuador | *Ipomoea* | *batatas* | 4X | + | - | - | - | - | + |
| PI 518474 |  | Mexico | *Ipomoea* | *batatas var. Apiculata* | 4X | + | + | + | + | + | - |
| PI 561246 | DLP 5282 |  | *Ipomoea* | *batatas* | 4X | + | - | - | - | - | - |
| PI 561247 | DLP 5283 |  | *Ipomoea* | *batatas* | 4X | + | - | - | - | - | + |
| PI 561248 | DLP 5284 |  | *Ipomoea* | *batatas* | 4X | + | - | - | - | - | + |
| PI 561261 |  |  | *Ipomoea* | *batatas* | 4X | + | - | - | - | - | + |

**TableN°3 List of *Ipomoea spp.* Series *Batatas* that were tested for the presence of *Ib*T-DNA1 and *Ib*T-DNA2 genes using degenerate primers**

Presence or absence was determined using the PCR reaction with degenerate primers (Table N° 6.1 and 6.2) for four ORFs on *Ib*T-DNA1 and one (ORF13) on *Ib*T-DNA2. ND indicates the test was not performed on the corresponding accessions. Positive samples were sequenced.

***Ipomoea trifida***

| **Accession number** | **Collecting number** | **Country of origin** | **Genus** | **Species** | **Ploidy**  **level** | **PCR MDH (Positive control)** | **PCR Dg *C-prot*** | **PCR Dg *Acs*** | **PCR Dg *IaaH*** | **PCR Dg *IaaM*** | | **PCR Dg *ORF13*** |
| --- | --- | --- | --- | --- | --- | --- | --- | --- | --- | --- | --- | --- |
| CIP 460097 | DLP 874 | Venezuela | *Ipomoea* | *trifida* | 2X | + | - | - | - | - | | - |
| CIP 460185 | DLP 3685 | Guatemala | *Ipomoea* | *trifida* | 2X | + | - | - | - | - | | + |
| CIP 460186 | DLP 3688 | Guatemala | *Ipomoea* | *trifida* | 2X | + | - | - | - | - | | + |
| CIP 460269 | DLP 3779 | Guatemala | *Ipomoea* | *trifida* | 2X | + | - | - | - | - | | - |
| CIP 460366 | DLP 3749 | Guatemala | *Ipomoea* | *trifida* | 2X | + | - | - | - | - | | - |
| CIP 460419 | DLP 4667 | Nicaragua | *Ipomoea* | *trifida* | 2X | + | - | - | - | - | | + |
| CIP 460425 | DLP 4674 | Nicaragua | *Ipomoea* | *trifida* | 2X | + | - | - | - | - | | + |
| CIP 460438 | DLP 4701 | Nicaragua | *Ipomoea* | *trifida* | 2X | + | - | - | - | - | | + |
| CIP 460534 | DLP 5348 | Cuba | *Ipomoea* | *trifida* | 2X | + | - | - | - | - | | - |
| CIP 460547 | DLP 5384 | Cuba | *Ipomoea* | *trifida* | 2X | + | - | - | - | - | | - |
| CIP 460021 | PI561544 | Venezuela | *Ipomoea* | *trifida* | 2X | + | - | - | - | - | | + |
| CIP 460135 | 540719 | Colombia | *Ipomoea* | *trifida* | 2X | + | - | - | - | - | | - |
| CIP 460111 | DLP 2997 | Colombia | *Ipomoea* | *trifida* | 2X | + | - | - | - | - | | - |
| CIP 460112 | DLP 2999 | Colombia | *Ipomoea* | *trifida* | 2X | + | - | - | - | - | | - |
| CIP 460132 | DLP 2988 | Colombia | *Ipomoea* | *trifida* | 2X | + | - | - | - | - | | - |
| CIP 460110 | DLP 2996 | Colombia | *Ipomoea* | *trifida* | 2X | + | - | - | - | - | | - |
| CIP 460108 | DLP 2992 | Colombia | *Ipomoea* | *trifida* | 2X | + | - | - | - | - | | - |
| CIP 460142 | DLP 5380 | Cuba | *Ipomoea* | *trifida* | 2X | + | - | - | - | - | | - |
| CIP 460143 | DLP 5386 | Cuba | *Ipomoea* | *trifida* | 2X | + | - | - | - | - | | - |
| CIP 460133 | DLP 2989 | Colombia | *Ipomoea* | *trifida* | 2X | + | - | - | - | - | | - |
| CIP 460120 | DLP 3012 | Colombia | *Ipomoea* | *trifida* | 2X | + | - | - | - | - | | - |
| CIP 460109 | DLP 2994 | Colombia | *Ipomoea* | *trifida* | 2X | + | - | - | - | - | | - |
| CIP 460113 | DLP 3000 | Colombia | *Ipomoea* | *trifida* | 2X | + | - | - | - | - | | - |
| CIP 460134 | DLP 2991 | Colombia | *Ipomoea* | *trifida* | 2X | + | - | - | - | - | | - |
| CIP 430406 | WT 9 | Japan | *Ipomoea* | *trifida* | 2X | + | - | - | - | - | | - |
| CIP107665.9 |  |  | *Ipomoea* | *trifida* | 2X | + | - | - | - | - | + | |
| CIP 460545 | DLP 5379 | Cuba | *Ipomoea* | *trifida* | 2X | + | - | - | - | - | + | |
| CIP107665.19 |  |  | *Ipomoea* | *trifida* | 2X | + | - | - | - | - | + | |

***Ipomoea triloba***

| **Accession number** | **Collecting number** | **Country of origin** | **Genus** | **Species** | **Ploidy**  **level** | **PCR MDH (Positive control)** | **PCR Dg *C-prot*** | **PCR Dg *Acs*** | **PCR Dg *IaaH*** | **PCR Dg *IaaM*** | **PCR Dg *ORF13*** |
| --- | --- | --- | --- | --- | --- | --- | --- | --- | --- | --- | --- |
| CIP 460011 | DLP 741 | Venezuela | *Ipomoea* | *triloba* | 2X | + | - | - | - | - | - |
| CIP 460100 | DLP 1281 | Ecuador | *Ipomoea* | *triloba* | 2X | + | - | - | - | - | - |
| CIP 460044 | DLP 1895 | Colombia | *Ipomoea* | *triloba* | 2X | + | - | - | - | - | - |
| CIP 460052 | DLP 2837 | Venezuela | *Ipomoea* | *triloba* | 2X | + | - | - | - | - | - |
| CIP 460012 | DLP 748 | Venezuela | *Ipomoea* | *triloba* | 2X | + | - | - | - | - | - |
| CIP 460526 | DLP 5334 | Cuba | *Ipomoea* | *triloba* | 2X | + | - | - | - | - | - |
| CIP 460558 | DLP 5426 | Peru | *Ipomoea* | *triloba* | 2X | + | - | - | - | - | - |
| CIP 460658 | DLP 1680 | Colombia | *Ipomoea* | *triloba* | 2X | + | - | - | - | - | - |
| CIP 460101 | DLP 2057 | Colombia | *Ipomoea* | *triloba* | 2X | + | - | - | - | - | - |
| CIP 460090 | DLP 2982 | Dominic Republic | *Ipomoea* | *triloba* | 2X | + | - | - | - | - | - |
| CIP 460116 | DLP 3003 | Colombia | *Ipomoea* | *triloba* | 2X | + | - | - | - | - | - |
| CIP 460554 | DLP 5405 | Mexico | *Ipomoea* | *triloba* | 2X | + | - | - | - | - | - |
| GRIF 6174 |  | Mexico | *Ipomoea* | *triloba* | 2X | + | - | - | - | - | - |
| PI 536044 |  | Mexico | *Ipomoea* | *triloba* | 2X | + | - | - | - | - | - |

***Ipomoea cordatotriloba***

| **Accession number** | **Collecting number** | **Country of origin** | **Genus** | **Species** | **Ploidy**  **level** | **PCR MDH (Positive control)** | **PCR Dg *C-prot*** | **PCR Dg *Acs*** | **PCR Dg *IaaH*** | **PCR Dg *IaaM*** | **PCR Dg ORF13** |
| --- | --- | --- | --- | --- | --- | --- | --- | --- | --- | --- | --- |
| CIP 460811 | AJ 62-96 | USA | *Ipomoea* | *Cordatotriloba** | 2X | + | - | - | - | - | - |
| CIP 460815 | AJ 67-39 | USA | *Ipomoea* | *Cordatotriloba** | 2X | + | - | - | - | - | - |
| CIP 460816 | AJ 67-43 | USA | *Ipomoea* | *cordatotriloba* | 2X | + | - | - | - | - | - |
| CIP 460785 | DLP 4014 | Argentina | *Ipomoea* | *cordatotriloba* | 2X | + | - | - | - | - | - |
| PI 518494 |  | Mexico | *Ipomoea* | *cordatotriloba* | 2X | + | + | + | + | + | - |

* Not true to type

***Ipomoea leucantha***

| **Accession number** | **Collecting number** | **Country of origin** | **Genus** | **Species** | **Ploidy**  **level** | **PCR MDH (Positive control)** | **PCR Dg *C-prot*** | **PCR Dg *Acs*** | **PCR Dg *IaaH*** | **PCR Dg *IaaM*** | **PCR Dg ORF13** |
| --- | --- | --- | --- | --- | --- | --- | --- | --- | --- | --- | --- |
| CIP 460104 | DLP 2112 | Colombia | *Ipomoea* | *leucantha* | 2X | + | - | - | - | - | - |
| CIP 460002 | DLP 431 | Ecuador | *Ipomoea* | *Leucantha** | 2X | + | - | - | - | - | - |
| PI 518481 | DLP2931 |  | *Ipomoea* | *leucantha* |  | + | - | - | - | - | - |
| PI 536036 |  |  | *Ipomoea* | *leucantha* |  | + | - | - | - | - | - |
| PI 540732 | 460115 |  | *Ipomoea* | *leucantha* |  | + | - | - | - | - | - |

* Not true to type

***Ipomoea tiliácea***

| **Accession number** | **Collecting number** | **Country of origin** | **Genus** | **Species** | **Ploidy**  **level** | **PCR MDH (Positive control)** | **PCR Dg *C-prot*** | **PCR Dg *Acs*** | **PCR Dg *IaaH*** | **PCR Dg *IaaM*** | **PCR Dg ORF13** |
| --- | --- | --- | --- | --- | --- | --- | --- | --- | --- | --- | --- |
| CIP 460397 | DLP 4638 |  | *Ipomoea* | *tiliacea** | 2X REV | + | - | - | - | - | - |
| CIP 460531 | DLP 4344 |  | *Ipomoea* | *tiliacea* | 4X | + | - | - | - | - | - |
| Butaud #3403 |  |  | *Ipomoea* | *tiliacea* |  | + | - | - | - | - | - |
| Butaud #3408 |  |  | *Ipomoea* | *tiliacea* |  | + | - | - | - | - | - |
| PI 165089 |  | Puerto Rico | *Ipomoea* | *tiliacea* |  | + | - | - | - | - | - |
| PI 518489 |  | Mexico | *Ipomoea* | *tiliacea* |  | + | - | - | - | - | - |
| CIP 460121 | DLP 3202 |  | *Ipomoea* | *tiliacea* | 4X | + | - | - | - | - | - |
| CIP 460145 | DLP 3186 |  | *Ipomoea* | *tiliacea* |  | + | - | - | - | - | - |

* Not true to type

***Ipomoea ramosissima***

| **Accession number** | **Collecting number** | **Country of origin** | **Genus** | **Species** | **Ploidy**  **level** | **PCR MDH (Positive control)** | **PCR Dg *C-prot*** | **PCR Dg *Acs*** | **PCR Dg *IaaH*** | **PCR Dg *IaaM*** | **PCR Dg ORF13** |
| --- | --- | --- | --- | --- | --- | --- | --- | --- | --- | --- | --- |
| CIP 460032 | DLP 1339 | Bolivia | *Ipomoea* | *ramosissima* | 2X | + | - | - | - | - | - |
| CIP 460036 | DLP 1656 | Bolivia | *Ipomoea* | *ramosissima* |  | + | - | - | - | - | - |
| CIP 460005 | DLP 657 | Peru | *Ipomoea* | *ramosissima* | 2X | + | - | - | - | - | - |
| CIP 460033 | DLP 1610 | Bolivia | *Ipomoea* | *ramosissima* | 2X | + | - | - | - | - | - |
| CIP 460006 | DLP 671 | Peru | *Ipomoea* | *ramosissima* | 2XREV | + | - | - | - | - | - |
| PI 540711 |  | Colombia | *Ipomoea* | *ramosissima* |  | + | - | - | - | - | - |
| PI 552786 |  | Bolivia | *Ipomoea* | *ramosissima* |  | + | - | - | - | - | - |

***Ipomoea grandifolia***

| **Accession number** | **Collecting number** | **Country of origin** | **Genus** | **Species** | **Ploidy**  **level** | **PCR MDH (Positive control)** | **PCR Dg *C-prot*** | **PCR Dg *Acs*** | **PCR Dg *IaaH*** | **PCR Dg *IaaM*** | **PCR Dg ORF13** |
| --- | --- | --- | --- | --- | --- | --- | --- | --- | --- | --- | --- |
| CIP 460189 | DLP 4037 | Argentina | *Ipomoea* | *grandifolia* | 2X | + | - | - | - | - | - |
| CIP 460190 | DLP 4160 | Paraguay | *Ipomoea* | *grandifolia* | 2X | + | - | - | - | - | - |
| CIP 460786 | DLP 4031 | Argentina | *Ipomoea* | *Grandifolia** | 2X | + | - | - | - | - | - |
| PI 561549 |  | Peru | *Ipomoea* | *grandifolia* |  | + | - | - | - | - | - |
| CIP 460106 | DLP 4921 | Argentina | *Ipomoea* | *grandifolia* |  | + | - | - | - | - | - |

* Not true to type

***Ipomoea splendor-sylvae* (*umbraticola*)**

| **Accession number** | **Collecting number** | **Country of origin** | **Genus** | **Species** | **Ploidy**  **level** | **PCR MDH (Positive control)** | **PCR Dg *C-prot*** | **PCR Dg *Acs*** | **PCR Dg *IaaH*** | **PCR Dg *IaaM*** | **PCR Dg ORF13** |
| --- | --- | --- | --- | --- | --- | --- | --- | --- | --- | --- | --- |
| CIP 460801 | DLP 4514 | Honduras | *Ipomoea* | *splendor-sylvae* |  | + | - | - | - | - | - |
| PI 561557 |  |  | *Ipomoea* | *splendor-sylvae* |  | + | - | - | - | - | - |

***Ipomoea sp.***

| **Accession number** | **Collecting number** | **Country of origin** | **Genus** | **Species** | **Ploidy**  **level** | **PCR MDH (Positive control)** | **PCR Dg *C-prot*** | **PCR Dg *Acs*** | **PCR Dg *IaaH*** | **PCR Dg *IaaM*** | **PCR Dg ORF13** |
| --- | --- | --- | --- | --- | --- | --- | --- | --- | --- | --- | --- |
| CIP 460250 | NCSU 11 | USA | *Ipomoea* | *Trifida** | 2X | + | + | + | + | + | - |
| CIP 460814 | AJ 67-38 | USA | *Ipomoea* | *Cordatotriloba** | 2X | + | + | + | + | + | - |

* Not true to type

**Other *Ipomoea* *spp*. from the series *Batatas.***

| **Accession number** | **Collecting number** | **Country of origin** | **Genus** | **Species** | **Ploidy**  **level** | **PCR MDH (Positive control)** | **PCR Dg *C-prot*** | **PCR Dg *Acs*** | **PCR Dg *IaaH*** | **PCR Dg *IaaM*** | **PCR Dg ORF13** |
| --- | --- | --- | --- | --- | --- | --- | --- | --- | --- | --- | --- |
| CIP 460824 | PI 518479 |  | *Ipomoea* | *tabascana* | 4X | + | - | - | - | - | - |
| PI 553012 |  |  | *Ipomoea* | *tenuissima* | 2X | + | + | + | + | + | - |
| PI 573335 |  |  | *Ipomoea* | *littoralis* | 2X | + | - | - | - | - | - |
| PI 634785 |  |  | *Ipomoea* | *lacunosa* |  | + | - | - | - | - | - |
| PI 549093 |  |  | *Ipomoea* | *cynanchifolia* |  | + | - | - | - | - | - |

**Table N°4 List of *Ipomoea* spp. (aside from *Batatas*) and other related genus tested for the presence of *Ib*T-DNA1 and *Ib*T-DNA2 genes using degenerate primers**

Presence or absence was determined using the PCR reaction with primers (Table N° 5.1 and 5.2) specific for four ORFs on *Ib*T-DNA1 and one (*ORF13*) on *Ib*T-DNA2. ND indicates the test was not performed on the corresponding accessions.

| **Accession number** | **DLP code** | **Country of origin** | **Genus** | **Species name** | **Ploidy** | **PCR MDH (Positive control)** | **PCR Dg *C-prot*** | **PCR Dg *Acs*** | **PCR Dg *IaaH*** | **PCR Dg *IaaM*** | **PCR Dg ORF13** |
| --- | --- | --- | --- | --- | --- | --- | --- | --- | --- | --- | --- |
| 587177 |  |  | *Ipomoea* | *hederifolia* |  | + | - | - | - | - | - |
| 561555 |  |  | *Ipomoea* | *quamoclit* |  | + | - | - | - | - | - |
| 664244 |  |  | *Calystegia* | *longipipes* |  | - | - | - | - | - | - |
| 657979 |  |  | *Xenostegia* | *tridentata* |  | + | - | - | - | - | - |
| 538281 |  |  | *Operculina* | *aequisepala* |  | + | - | - | - | - | - |
| GRIF6144 |  |  | *Merremia* | *diseccta* |  | + | - | - | - | - | - |
| GRIF6286 |  |  | *Merremia* | *quinquefolia* |  | + | - | - | - | - | - |

**TableN°5 Primers used for PCR**

| **5.1 Primers specific used for PCR** | | |
| --- | --- | --- |
| **Target region** | **Description** | **Sequence** |
| *IaaM* | Forward Primer | ACTCCAGACGATCTTAGCCACTTC |
|  | Reverse Primer | CATTCTCCAGCACTCCTCCTG |
| *C-protein* | Forward Primer | GATCAGTACGAACGCAAGCAAG |
|  | Reverse Primer | CGTATATCGTTGAAGAAATGGAC |
| *IaaH* | Forward Primer | CGGCAAACCGTCTGATGTGA |
|  | Reverse Primer | AGTCCAAACCCTAGAAGCCCGAT |
| *Acs* (agrocinopine synthase) | Forward Primer | AGTCAGCGATTGCAGCGGTA |
|  | Reverse Primer | GCGTCCCTATCGTTTCCCA |
| *RolB/RolC* | Forward Primer | CTCAATGTAGCTCAGAACGGTTACG |
|  | Reverse Primer | TCTTAGTGTGGCATCCTACTCTATGT |
| *Orf17n* | Forward Primer | AGCGTGATCGACAACATCTCAT |
|  | Reverse Primer | TGAGCGAACCTCTTGCATACT |
| *Orf18/Orf17n* | Forward Primer | TGACCAAATTCCAAGGTTCCAGC |
|  | Reverse Primer | ATCCGTATCAGCGTGAACTTCATG |
| *Orf13* | Forward Primer | TGACAGAGGGATGGCGGATG |
|  | Reverse Primer | ACTGGAGGTATTGCGTTCGGAT |
| *MDH* (Malate Dehydrogenase) | MDH-H968 (Forward Primer) | GCATCTGTGGTTCTTGCAGG |
|  | MDH-C1163 (Reverse Primer) | CCTTTGAGTCCACAAGCCAA |
| **5.2 Degenerate Primers used for PCR** | | |
| **Target region** | **Description** | **Sequence** |
| *IaaM* | Forward Primer | GARATGGGRGCSATGCGWTTTC |
|  | Reverse Primer | TCRRTAAASCCRCTTTCAAADAC |
| *C-prot* | Forward Primer | CADASKCARGAAATGMGGTGGC |
|  | Reverse Primer | CGGCRGTTGCSGTGAAMAGRCG |
| *IaaH* | Forward Primer | TTRCTGCTKGGRTCBACRTTYCGC |
|  | Reverse Primer | CCGRTNAGCCCYACCCGRGAC |
| *Acs* (agrocinopine synthase) | Forward Primer | GAYYTMAAYWCYTGGCGRRTITC |
|  | Reverse Primer | CCYAGAWWGMCATYTCATCYTC |
| *Orf13* | Forward Primer | ATYCMSTCCAGYCTYCGHTTTC |
|  | Reverse Primer | CGCATBMGAGCCTGYAAMAG |
| **5.3 Primers used for PCR (Flanking region)** | | |
| **Target region** | **Description** | **Sequence** |
| Flanking region *Ib*T-DNA1 | Forward Primer | GCAGCCATGCAAGAAGGTATG |
|  | Reverse Primer | CACACGATCTTCACATCTCGGA |
| Flanking region *Ib*T-DNA2 | Forward Primer | CAGGTGTTACTCAAGTGGACTTCTC |
|  | Reverse Primer | ATAGCCGCAACGAACGCCGAT |
| *F-box* gene | Forward Primer | TCCGAGATGTGAAGATCGTGTG |
|  | Reverse Primer | GTTTGGAATATAGTCAAGAATGCTG |
| *UcpB* gene | Forward Primer | CAATAGCAGGCACTGTAAGCAC |
|  | Reverse Primer | CGAGGTGAGTAGGAGCAGTTCTTA |
| **5.4 PCR primers to amplify probes used in Southern blotting** | | |
| **Target region** | **Description** | **Sequence** |
| *C-protein* | Forward Primer | CTTTGCGACTCATCCAACACGT |
|  | Reverse Primer | AATCCACTCTGTCCTTCTCGGA |
| Flanking region | Forward Primer | GCACACGATCTTCACATCTCG |
|  | Reverse Primer | GCAGCCATGCAAGAAGGTATG |
| *Orf17n* | Forward Primer | AGCGTGATCGACAACATCTCAT |
|  | Reverse Primer | TGAGCGAACCTCTTGCATACT |

**Table N°6 Identity values for *Ib*T-DNA1 genes among *Ipomoea* spp. and *I. batatas* CIP 420065**

| ***Ipomoea species*** | **Identity Values (%)** | | | |
| --- | --- | --- | --- | --- |
|  | **Acs** | **C-prot** | **iaaH** | **iaaM** |
| *Ipomoea batatas* (L.) Lam var Apiculata PI 518474 (4x) | 99.5 | 99.9 | 100 | 99.5 |
| *Ipomoea batatas* (L.) Lam CIP 460577 (4x) | 99.5 | 99.9 | 99.8 | 99.5 |
| *Ipomoea batatas* (L.) Lam CIP 430434 (4x) | 99.7 | 99.7 | 99.7 | 100 |
| *Ipomoea batatas* (L.) Lam CIP 403270 (4x) | - | - | - | 99.8 |
| *Ipomoea cordatotriloba* CIP 460814 | 98.3 | 96.2 | 97 | 97.9 |
| *Ipomoea cordatotriloba* PI 518494 | 98.3 | 96.3 | 97 | 97.9 |
| *Ipomoea tenuissima* PI 553012 | 98.5 | 97 | 97 | 97.5 |
| *Ipomoea triloba* CIP 460250 | 98.2 | 96.3 | 97 | 97.9 |

**Table N°7 Identity values for *Ib*T-DNA genes among *Ipomoea* spp. and *I. batatas* CIP 420065**

| ***Ipomoea species*** | **Identity Values (%)** |
| --- | --- |
|  | **ORF13** |
| *Ipomoea trifida* CIP 460425 | 96,3 |
| *Ipomoea trifida* CIP 460419 | 96.3 |
| *Ipomoea trifida* CIP107665.9 | 96.3 |
| *Ipomoea trifida* CIP 460185 | 96.1 |
| *Ipomoea trifida* CIP 460186 | 96.3 |
| *Ipomoea trifida* CIP 460545 | 96.3 |
| *Ipomoea trifida* PI 561544 | 99.1 |

**Table N°8 GenBank accession numbers**

| **C-prot gene** | | |
| --- | --- | --- |
| **Sequence_ ID** | **Specimen voucher** | **GenBank accession numbers** |
|  |  |  |
| Seq1 | CIP_440146 | MN129055 |
| Seq2 | Xu_781 | MN129056 |
| Seq3 | CIP_400450 | MN129057 |
| Seq4 | CIP_440116 | MN129058 |
| Seq5 | CIP_441724 | MN129059 |
| Seq6 | CIP_440032 | MN129060 |
| Seq7 | CIP_440398 | MN129061 |
| Seq8 | CIP_420065 | MN129062 |
| Seq9 | CIP_440132 | MN129063 |
| Seq10 | CIP_440031 | MN129064 |
| Seq11 | CIP_440166 | MN129065 |
| Seq12 | CIP_460577 | MN129066 |
| Seq13 | PI_518474 | MN129067 |
| Seq14 | CIP_460814 | MN129068 |
| Seq15 | PI_518494 | MN129069 |
| Seq16 | PI_553012 | MN129070 |
| Seq17 | CIP_460250 | MN129071 |
|  |  |  |
| **Acs gene** | | |
| **Sequence ID** | **Specimen voucher** | **GenBank accession numbers** |
|  |  |  |
| Seq1 | CIP_440031 | MN129072 |
| Seq2 | CIP_440032 | MN129073 |
| Seq3 | Xu781 | MN129074 |
| Seq4 | CIP_440398 | MN129075 |
| Seq5 | CIP_440166 | MN129076 |
| Seq6 | CIP_441724 | MN129077 |
| Seq7 | CIP_440132 | MN129078 |
| Seq8 | CIP_440116 | MN129079 |
| Seq9 | CIP_440146 | MN129080 |
| Seq10 | CIP_420065 | MN129081 |
| Seq11 | CIP_440450 | MN129082 |
| Seq12 | PI_518474 | MN129083 |
| Seq13 | CIP_460577 | MN129084 |
| Seq14 | CIP_460250 | MN129085 |
| Seq15 | CIP_460814 | MN129086 |
| Seq16 | PI_518494 | MN129087 |
| Seq17 | PI_553012 | MN129088 |
|  |  |  |
|  |  |  |
| **iaaH gene** | | |
| **Sequence ID** | **Specimen voucher** | **GenBank accession numbers** |
|  |  |  |
| Seq1 | CIP_440146 | MN129089 |
| Seq2 | Xu781 | MN129090 |
| Seq3 | CIP_440398 | MN129091 |
| Seq4 | CIP_440166 | MN129092 |
| Seq5 | CIP_440031 | MN129093 |
| Seq6 | CIP_440032 | MN129094 |
| Seq7 | CIP_440450 | MN129095 |
| Seq8 | CIP_440116 | MN129096 |
| Seq9 | CIP_420065 | MN129097 |
| Seq10 | CIP_440132 | MN129098 |
| Seq11 | CIP_441724 | MN129099 |
| Seq12 | CIP_460577 | MN129100 |
| Seq13 | PI_518474 | MN129101 |
| Seq14 | PI_553012 | MN129102 |
| Seq15 | CIP_460250 | MN129103 |
| Seq16 | PI_518494 | MN129104 |
| Seq17 | CIP_460814 | MN129105 |
|  |  |  |
| **ORF13 gene** | | |
| **Sequence ID** | **Specimen voucher** | **GenBank accession numbers** |
|  |  |  |
| Seq1 | CIP 440031 | MN159239 |
| Seq2 | CIP 440166 | MN159240 |
| Seq3 | CIP 420065 | MN159241 |
| Seq4 | CIP 440146 | MN159242 |
| Seq5 | CIP 440116 | MN159243 |
| Seq6 | CIP 441724 | MN159244 |
| Seq7 | CIP 440305 | MN159245 |
| Seq8 | Taizhong 6 | MN159246 |
| Seq9 | CIP 460577 | MN159247 |
| Seq10 | CIP 403552 | MN159248 |
| Seq11 | CIP 460578 | MN159249 |
| Seq12 | PI 561247 | MN159250 |
| Seq13 | PI 561248 | MN159251 |
| Seq14 | PI 561255 | MN159252 |
| Seq15 | PI 561258 | MN159253 |
| Seq16 | PI 561261 | MN159254 |
| Seq17 | CIP 460425 | MN159255 |
| Seq18 | CIP 460419 | MN159256 |
| Seq19 | CIP107665.19 | MN159257 |
| Seq20 | CIP107665.9 | MN159258 |
| Seq21 | CIP_460185 | MN159259 |
| Seq22 | CIP_460186 | MN159260 |
| Seq23 | CIP_460545 | MN159261 |
| Seq24 | PI_561544 | MN159262 |
|  |  |  |
| **iaaM gene** | | |
| **Sequence_ ID** | **Specimen voucher** | **GenBank accession numbers** |
|  |  |  |
| Seq1 | CIP 403270 | MN159263 |
| Seq2 | CIP 440032 | MN159264 |
| Seq3 | CIP 440166 | MN159265 |
| Seq4 | CIP 420065 | MN159266 |
| Seq5 | CIP 460577 | MN159267 |
| Seq6 | CIP 440274 | MN159268 |
| Seq7 | CIP 440398 | MN159269 |
| Seq8 | CIP 400450 | MN159270 |
| Seq9 | CIP 441724 | MN159271 |
| Seq10 | CIP 440116 | MN159272 |
| Seq11 | CIP 440146 | MN159273 |
| Seq12 | CIP 440031 | MN159274 |
| Seq13 | CIP Xu 781 | MN159275 |
| Seq14 | PI 518474 | MN159276 |
| Seq15 | CIP 430434 | MN159277 |
| Seq16 | PI 553012 | MN159278 |
| Seq17 | CIP 460814 | MN159279 |
| Seq18 | PI 518494 | MN159280 |
| Seq19 | CIP 460250 | MN159281 |
|  |  |  |
| **Flanking region IbT-DNA1 (Region F-box-IbT-DNA1)** | | |
| **Sequence_ ID** | **Specimen voucher** | **GenBank accession numbers** |
|  |  |  |
| Seq1 | Taizhong 6 | MN159282 |
| Seq2 | CIP 420065 | MN159283 |
| Seq3 | CIP 440166 | MN159284 |
| Seq4 | CIP 440031 | MN159285 |
| Seq5 | PI 518474 | MN159286 |
| Seq6 | CIP 440132 | MN159287 |
| Seq7 | CIP 460577 | MN159288 |
| Seq8 | CIP 403270 | MN159289 |
|  |  |  |
| **Flanking region IbT-DNA1 (Region F-box)** | | |
| **Sequence_ ID** | **Specimen voucher** | **GenBank accession numbers** |
|  |  |  |
| Seq1 | Taizhong 6 | MN159290 |
| Seq2 | CIP 420065 | MN159291 |
| Seq3 | CIP 403270 | MN159292 |
| Seq4 | PI 518494 | MN159293 |
| Seq5 | CIP 460250 | MN159294 |
|  |  |  |
| **Flanking region IbT-DNA2 (Region UcpB-IbT-DNA2)** | | |
| **Sequence_ ID** | **Specimen voucher** | **GenBank accession numbers** |
|  |  |  |
| Seq1 | PI 561544 | MN159295 |
| Seq2 | CIP 561247 | MN159296 |
| Seq3 | CIP 403552 | MN159297 |
| Seq4 | Taizhong 6 | MN159298 |
|  |  |  |
| **Flanking region IbT-DNA2 (Region UcpB gene)** | | |
| **Sequence_ ID** | **Specimen voucher** | **GenBank accession numbers** |
|  |  |  |
| Seq1 | CIP 561258 | MN159299 |
| Seq2 | CIP 561247 | MN159300 |
| Seq3 | Taizhong 6 | MN159301 |
|  |  |  |
